# Synthesis and Study of New Quinolineaminoethanols as Anti-Bacterial Drugs

**DOI:** 10.3390/ph12020091

**Published:** 2019-06-18

**Authors:** Pierre Laumaillé, Alexandra Dassonville-Klimpt, François Peltier, Catherine Mullié, Claire Andréjak, Sophie Da-Nascimento, Sandrine Castelain, Pascal Sonnet

**Affiliations:** 1 AGIR, EA 4294, UFR of Pharmacy, Jules Verne University of Picardie, 80037 Amiens, France; pierre.laumaille@etud.u-picardie.fr (P.L.); peltier.francois@chu-amiens.fr (F.P.); catherine.mullie@u-picardie.fr (C.M.); andrejak.claire@chu-amiens.fr (C.A.); sophie.da-nascimento@u-picardie.fr (S.D.-N.); sandrine.castelain@u-picardie.fr (S.C.); 2 Department of Bacteriology, Amiens University Hospital, 80054 Amiens, France; 3 Respiratory and Intensive Care Unit, University Hospital Amiens, 80054 Amiens, France

**Keywords:** Quinoline, tuberculosis, nosocomial infections, ESKAPEE bacteria, mycobacterium

## Abstract

The lack of antibiotics with a novel mode of action associated with the spread of drug resistant bacteria make the fight against infectious diseases particularly challenging. A quinoline core is found in several anti-infectious drugs, such as mefloquine and bedaquiline. Two main objectives were set in this work. Firstly, we evaluated the anti-mycobacterial properties of the previous quinolines **3**, which have been identified as good candidates against ESKAPEE (*Enterococcus faecium*, *Staphylococcus aureus*, *Klebsiella pneumoniae*, *Acinetobacter baumannii*, *Pseudomonas aeruginosa*, *Enterobacter* spp. and *Escherichia coli*) bacteria. Secondly, a new series **4** was designed and assessed against the same bacteria strains, taking the pair of enantiomers **3m**/**3n** as the lead. More than twenty compounds **4** were prepared through a five-step asymmetric synthesis with good enantiomeric excesses (>90%). Interestingly, all compounds of series **3** were efficient on *M. avium* with MIC = 2–16 µg/mL, while series **4** was less active. Both series **3** and **4** were generally more active than mefloquine against the ESKAPEE bacteria. The quinolines **4** were either active against Gram-positive bacteria (MIC ≤ 4 µg/mL for **4c**–**4h** and **4k**/**4l**) or *E. coli* (MIC = 32–64 µg/mL for **4q**–**4v**) according to the global lipophilicity of these compounds.

## 1. Introduction

Infectious diseases are the second most prevalent cause of death in the world. Among them, nosocomial infections and tuberculosis are particularly worrying. Approximatively 15% of all hospitalized patients suffer from healthcare associated infections [1]. ESKAPEE bacteria (*Enterococcus faecium*, *Staphylococcus aureus*, *Klebsiella pneumoniae*, *Acinetobacter baumannii*, *Pseudomonas aeruginosa*, *Enterobacter* spp. and *Escherichia coli*) are the most common pathogens involved in nosocomial infections and their antibiotic resistances make it difficult to implement effective treatments. Mycobacteria, such as *Mycobacterium tuberculosis*, which is responsible for tuberculosis (TB), are also becoming resistant to anti-TB drugs used for standard therapy. TB is one of the most common infectious diseases. In 2017, 10 million people developed TB infection and 1.6 million have died due to this disease. 400,000 cases of multi-drug resistant (MDR) TB and 40,000 cases of extensively-drug resistant (XDR) TB were reported in 2018 [2]. The treatment of uncomplicated TB is already difficult because it requires a combination of four anti-mycobacterial drugs such as ethambutol, rifampin, isoniazid and pyrazinamide for two months completed by the addition of rifampin and isoniazid across four additional months [3]. The cure of MDR and XDR TB involves more drugs, more time (sometimes 24 months) and is much more expensive [4]. Non-Tuberculosis Mycobacteria (NTM) such as *M. avium* and *M. abscessus* are considered as emerging pathogens and are also worrisome. In some countries, NTM pulmonary infections appear to be more frequent than TB [5]. The treatment of NTM pulmonary infections involves a combination of at least two anti-mycobacterial drugs according to the severity of the disease and the tolerance to the cure. For example, the treatment of a clarithromycin-sensitive *M. avium* complex lung infection requires an association of ethambutol, rifampin and clarithromycin, which must be continued for at least 12 months after culture conversion [6].

So, it is evident that ESKAPEE bacteria and mycobacteria are a major problem for public health due to their resistances to the antibiotics. Unfortunately, since 1970 few antibiotics with new mechanisms of action have been marketed. They are either active against Gram-positive bacteria or mycobacteria but not against gram-negative bacteria. Consequently, there is a dire need to design new drugs efficient against a larger panel of bacteria and able to fight resistant strains.

**Figure 1:**
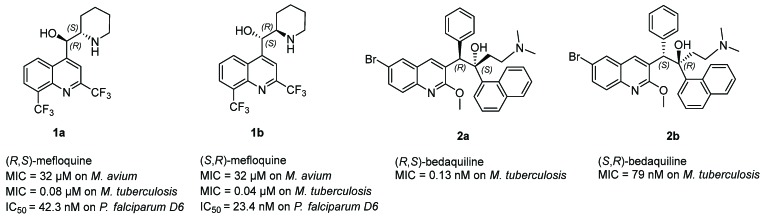
Mefloquine **1** and bedaquiline **2** enantiomers and their biological activities.

The quinoline core is found in anti-infectious drugs such as mefloquine (MQ **1a/1b**, Figure 1) or bedaquiline (BQ, compound **2a**, Figure 1) [7,8,9,10,11,12,13]. BQ **2** is a diarylquinoline which was recently marketed as an anti-TB compound to treat MDR *M. tuberculosis* [7], while MQ **1** is an aminoquinolinemethanol used as antimalarial drug [10]. These two drugs, which possess two asymmetric centers, are used either as a racemic mixture ((*R*,*S*)-MQ **1a** + (*S*,*R*)-MQ **1b**) or as enantiopure form ((*R*,*S*)-BQ **2a**). Despite their structural similarity, these two quinolines possess two different antimicrobial spectra. BQ **2a** is only active against mycobacteria [7,14] in the nanomolar range, while MQ **1a/1b** is active against *Plasmodium falciparum* in the nanomolar range and Gram-positive bacteria and mycobacteria in the micromolar range (Figure 1) [9,15,16,17]. (*R*,*S*)-BQ **2a** targets very specific mycobacteria, including *M. tuberculosis* and *M. avium* [14], due to its great affinity to the mycobacteria F0F1-ATP synthase [18]. This one was reported to be about 630-fold more active than its enantiomer **2b** against *M. tuberculosis* [19]. The mechanism of action in MQ **1** is much less clear. Despite its use as an antimalarial since the 1970s, no study has clearly highlighted the main antiplasmodial and/or antibacterial MQ-target [9,20,21,22]. However, some biochemical evidence has associated the antibacterial properties of MQ to its interaction with the F0F1-ATP synthase [21]. Even if MQ **1a/1b** is used as racemic mixture, studies suggest this is the best activity of the enantiomer (+)-(*S*,*R*)-MQ compared to its optical antipode against *P. falciparum* and *M. tuberculosis* in vitro and *M. avium* in vivo [16,17,23]. However, this difference in activity is not observed against bacteria such as *S. aureus*, *Enterococcus faecalis* or *Streptococcus pneumonia* [9,21].

**Figure 2:**
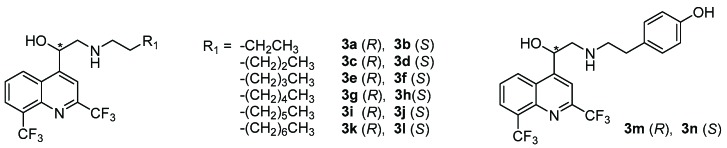
Previous series of quinoline-based antimicrobial drugs **3a**–**3n**.

During previous work on the development of new enantiopure MQ-based antimalarial drugs, we identified a series of compounds **3** containing an aliphatic side chain (Figure 2) [22]. These MQ analogs **3a**–**3l** displayed activities on the nanomolar range against *P. falciparum* 3D7 and W2 strains whatever the length of the aliphatic side chain while the enantiomers **3m** and **3n** were inactive on these strains (half maximal inhibitory concentration, IC_50_ > 400 nM) [22]. Interestingly, for **3a**–**3l**, (*S*)-enantiomers were always more active than their (*R*)-counterpart by a factor of two to 15.

Thereafter, the antibacterial potential of all these compounds was assessed on Gram-positive strains (*S. aureus* and *E. faecalis*) and Gram-negative strains (*E. coli* and *P. aeruginosa*) (Table 1) [24]. The MQ analogs **3** were often more active against Gram-positive bacteria compared to Gram-negative bacteria. However, some of them (**3c**, **3f, 3m** and **3n**) showed a minimum inhibitory concentration (MIC) lower than 32 µg/mL on *E. coli*. For this series, the side chain length has an influence on the antibacterial activity in connection with the lipophilicity determined here by the clogP value. Compounds displaying a clogP value ranging from 4.95 (**3e**/**3f**) to 5.70 (**3i**/**3j**) were more active against Gram-positive bacteria. The best activity against Gram-negative bacteria was observed for compounds with a clogP value lower than 4.60 corresponding to C4 (**3a**/**3b**) and C5 (**3c**/**3d**) alkyl side chain length or a 4-hydroxyphenylethyl group (**3m**/**3n).** This latter pair of enantiomers was the more effective on *E. coli* (MIC = 8 (**3m**) and 16 (**3n**) µg/mL) while remaining active on Gram-positive bacteria (MIC = 8 µg/mL).

**Figure 3:**
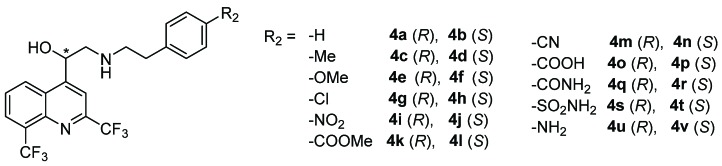
Novel series of quinoline-based drugs **4**.

In light of these encouraging results, we are particularly interested in establishing novel structure-antimicrobial activity relationships in the promising quinoline-based series **3.** The **3m/3n** pair displaying the broader antimicrobial spectrum of this series possesses a phenyl grafted to the alkyl side chain. It allows us to make structural changes to determine new structure-activity relationships. Here, the chosen optimization strategy consists of exploring the hydrophobic (π-constant) and the electronic space (σ) around the phenyl by modifying the R_2_ group to form a novel series **4** of quinoline-based drugs (Figure 3). The substituents (R_2_) were selected on the base of the Craig Plot tool to ensure that all possible combinations of pi (π) and sigma (σ) were studied [25].

We present herein not only the antimycobacterial activity of the previously synthetized quinolineaminoethanols **3**, but also the synthesis, antibacterial activities of the new enantiopure quinoline-based drugs **4**, and analogs of the compounds **3m**/**3n**. We will compare the antibacterial activities of these two series **3** and **4** in relation to the nature of their quinoline substituents and their stereochemistry to establish structure–activity relationships.

## 2. Results and Discussion

### 2.1. Chemical Synthesis

**Scheme 1:**
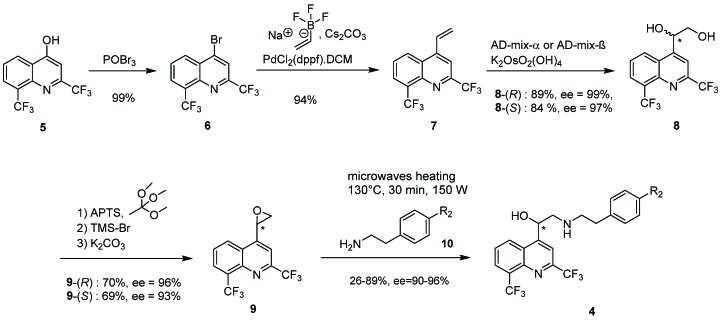
Synthetic pathway of compounds **4.**

The synthesis of compounds **4** was carried out from the 4-hydroxyquinoline **5** in a five-step (Scheme 1). We have previously described the first four steps that allowed us to obtain the key intermediate **9** [26]. First, the 4-hydroxyquinoline **5** was reacted with POBr_3_ to create **6**, which was coupled with the sodium vinyl trifluoroborate through a Suzuki reaction to give the 4-vinylquinoline **7** in 94% yield. Then, an asymmetric Sharpless dihydroxylation was carried out using either AD-mix-α to give the diol **8** as the *S* enantiomer or AD-mix-β to obtain **8** as the *R* enantiomer. A three-step condensation with retention of the configuration allowed us to obtain the two enantiomers of the key epoxyde **9** depending of the diol used. Finally, (*R*)-**9** or (*S*)-**9** was reacted with the commercially available amines **10** to give the corresponding compound **4**. Only the amines **10a** (R_2_ = COOMe) and **10b** (R_2_ = CONH_2_) allowed us to obtain the two pairs of enantiomers, and **4k**/**4l** and **4q**/**4r**, respectively were synthesized in one step (see experimental Section 3.2.1.).

Hence, twenty-two compounds **4** were obtained, in five steps with a global yield ranging from 14 to 47% (Table 2). The enantiomeric purity was quantified using chiral HPLC (High Performance Liquid Chromatography) where possible and the optical rotation was measured for each couple of enantiomers (Table 2). All ^1^H and ^13^C NMR spectra of compounds **4a**–**4u** are provided in Supplementary Materials (Figures S1–S22).

### 2.2. Biological Activity

MQ and compounds **4** were tested *in vitro* against *S. aureus* CIP 103.429 and *E. faecalis* CIP 103.214 (CIP: Collection de l’Institut Pasteur, Paris, France) as Gram-positive bacteria and *E. coli* DSM 1103 and *P. aeruginosa* DSM 1117 as Gram-negative bacteria (Deutsche Sammlung für Mikroorganismen, Braunschweig, Germany). The anti-mycobacterial activity of compounds **3** and **4** was also assessed through assays on *M. avium* ATCC 700,898 (American Type Culture Collection) and compared with those of MQ and its enantiomers **1a** and **1b**. The MIC determinations were carried out using the broth microdilution technique as advised by the Clinical and Laboratory Standards Institute (CLSI) with drug concentrations ranging from 0.0625 to 128 µg/mL [27] for the anti-bacterial screening and from 2 to 64 µg/mL for the anti-mycobacterial evaluation [28]. *M. avium* susceptibility test was realized in cation-adjusted Mueller–Hinton broth (CAMHB) supplemented with Oleic Albumin Dextrose Catalase (OADC). Furthermore, as anti-TB drugs such as clarithromycin can be influenced by the growth medium, MiddleBrook 7H9 (MB 7H9) supplemented with 5% OADC was used in parallel. Ciprofloxacin was used as control for Gram-negative and Gram-positive bacteria while clarithromycin was employed for experiments with mycobacteria. In this latter assay, rifampin, ethambutol, ciprofloxacin and bedaquiline **2a** were added as indicators.

As for the first series **3**, the quinolines **4** were generally more active against Gram-positive bacteria than Gram-negative bacteria (Table 3 vs. Table 1). A better activity was often observed on *S. aureus* compared to *E. faecalis*, especially for the pairs of enantiomers **4i**/**4j** and **4m**/**4n** carrying the more electron-withdrawing groups (σp = 0.778 (R_2_ = NO_2_) and 0.660 (R_2_ = CN)). The additional four efficiency pairs of enantiomers **4c**/**4d**, **4e/4f**, **4g/4h** and **4k/4l** on this strain (MIC ≤ 4 µg/mL) were among the more lipophilic with a clogP values between 4.37 (**4k**/**4l**) and 5.75 (**4g**/**4h**). These were less active than the referenced ciprofloxacin but more active than MQ by four to 16-fold depending on the substituent. The same trend was found on *E. faecalis* even if **4e**/**4f** and **4k** displayed a slightly superior MIC (MIC = 8 µg/mL).

As expected, the most hydrophilic compounds **4o**–**4v** (R_2_ = COOH, CONH_2_, SO_2_NH_2_, NH_2_), were less active against Gram-positive strains (MIC between 8 and 64 µg/mL) but showed a better activity than others compounds of this series, except **4o–4p** with a COOH group, against *E. coli* (MIC between 32 and 64 µg/mL). These three pairs of enantiomers **4q–4v** displayed an activity similar or better (MIC = 8–64 µg/mL) than MQ (MIC = 64 µg/mL) against *E. coli* but lower than the lead pair of enantiomers **3m/3n** (MIC = 8–16 µg/mL). For all quinolines **4**, no significant difference in anti-bacterial activities was observed between the enantiomers.

Among the quinolines of this series **4**, the more active on *E. coli* **4q**–**4v** (R_2_ = CONH_2_, SO_2_NH_2_, NH_2_) were also more effective against *M. avium* with an MIC equal to 64 µg/mL. Not surprisingly, the pair of enantiomers **3m/3n** (R_2_ = OH) were the most efficient on this mycobacterium with an MIC nearer to those of ethambutol and rifampin. However, the compounds **3a**-**3l** with aliphatic side chains were generally more active than the compounds **4** with MIC ≤ 32 µg/mL and often equal to 2 or 4 µg/mL, especially in the CAMHB. For this series **3**, the anti-mycobacterial activity seems less dependent to the side chain length and to the clogP values (4.30–6.01) compared to the antibacterial activity (Table 4 vs. Table 1). The quinolines **3** were often less active than the reference clarithromycin in CAMHB medium but as active in MB 7H9 medium, as active as MQ and more active than the three anti-TB drugs; ethambutol, rifampin, and ciprofloxacin. A slight difference of activity between the two enantiomers can be observed especially for the pair **3g/3h** in the MB 7H9 culture medium.

Thanks to these results, it is possible to establish new structure-activity relationships about the two series of quinoline-based drugs **3** and **4** (Table 5). For the quinolines **4** and **3m**/**3n** carrying a phenyl group, the compounds with a clogP value above four were the more active against Gram-positive bacteria. Although we do not know the mechanism of action of this quinoline series, this better activity against Gram-positive bacteria perhaps could be due to a better intracellular internalization. None of the quinolines (series **3** and **4**) were active against *P. aeruginosa* but some of them showed a moderate activity on *E. coli*. The compounds with a clogP value below 4 (**4q**–**4t**) were the less efficient against Gram-positive strains but were active against *E. coli* and *M. avium*, except if the compound was a carboxylic acid (**4o**–**4p**). The introduction of a more polar substituent of the phenyl core may allow a better translocation through the outer membrane of the Gram-negative bacteria. For clogP value ranging from four to five, the best activities against *E. coli* and *M. avium* were observed for the compounds carrying hydrophilic substituents (-π) and preferably with an electron-donor effect (-σ) around the phenyl group (**3m**/**3n**, **4u**/**4v**). For the quinoline-based drugs with an aliphatic side chain (**3a**–**3l**), all the compounds were active against *M. avium* whatever the side chain length (MIC ≤ 16 µg/mL in CAMHB).

## 3. Materials and Methods

### 3.1. Generalities

All reagents and solvents were obtained from commercial suppliers and were used without further purification. Anhydrous solvents were dried with the solvent drier, Pure Solv-Innovative Technology PS-MD-5. Some reactions were carried out with a Discover SP microwave reactor (commercialized by CEM). Column chromatography was performed over silica gel Kielselgel 60 (40–60 µm). Routine monitoring of reactions was carried out using Merck Silica Gel 60 F254 plates thin layer chromatography (TLC) and visualized under UV light (254 nm). Nuclear magnetic resonance (NMR) spectra were recorded using Bruker 400-cryosonde NMR instrument (^1^H NMR at 400 MHz and ^13^C NMR at 101 MHz). Chemical shifts were expressed in parts per million (ppm) downfield from tetramethylsilane and were referenced to the deuterated solvent. ^1^H NMR and ^13^C NMR data were reported in the order of chemical shift, multiplicity (s = singlet, d = doublet, t = triplet, q = quartet, qt = quintuplet, m = multiplet), integration, coupling constants in Hertz (Hz). LC-HRMS analyses were performed on an ACQUITY UPLC H-Class system (Waters-Micromass, Manchester, UK) coupled with a SYNAPT G2-Si Q-TOF hybrid quadrupole time-of-flight instrument (Waters-Micromass, Manchester, UK), equipped with an electrospray (ESI) ionization source (Z-spray). Enantiomeric excesses were measured with Schimadzu LC-20AD equipped with a Chiralpak column (IA, IB, IC, ID or IG). Specific rotations were measured on a Jasco P1010 polarimeter, with 1 dm length cuvette and a continuous wave lamp at sodium D-line (589 nm). Concentrations were expressed in g/100 mL and measures were realized in dichloromethane at 23 or 26 °C. High-resolution mass spectra were obtained from a Micromass-Waters Q–TOF Ultima spectrometer, in electrospray ionization mode (positive or negative). Infrared spectra were recorded with a Jasco FT/IR-4200 and were reported using the frequency of absorption (cm^−1^). Gas Chromatography–Mass Spectrometry (GCMS) was carried out on a Schimadzu GCMS-QP2010S equipped with a SLB-5 ms column. Melting points were determined with a Stuart SMP3 device.

### 3.2. Synthesis

The synthetic route to prepare **9** has already been described by our research group [26].

#### 3.2.1. Synthesis of amines **10**

##### Methyl 4-(2-aminoethyl)benzoate **10a**

4-(2-aminoethyl)benzoic acid hydrochloride (250 mg, 1 eq) was solved in MeOH (5 mL). SOCl_2_ (310 mg, 2.1 eq) was added slowly at 0 °C and the mixture was stirred at 65 °C over 10 h. The crude product was evaporated to dryness then solved in dichloromethane (DCM) and evaporated again. This procedure was repeated three times. An aqueous NaOH solution (1M) was added, and the mixture was extracted three times with DCM. Then, the saturated NaCl solution was added and the mixture was extracted three times with DCM. The combined organic layers were dried over Na_2_SO_4_ and concentrated in vacuo to give 214 mg of yellow oil (97% yield). ν_max_: 3280, 2947, 2850, 1717, 1628, 1542, 1436, 1279, 1183, 1108, 764, 632 cm^−1^; ^1^H NMR (400 MHz, Chloroform-*d*) *δ* 7.97 (d, ^3^*J* = 8.5 Hz, 2H), 7.29 (m, 2H), 3.91 (s, 3H), 3.00 (t, ^3^*J* = 6.8 Hz, 2H), 2.81 (t, ^3^*J* = 6.8 Hz, 2H). ^13^C NMR (101 MHz, Chloroform-*d*) *δ* 168.0, 145.4, 129. 8, 128.9, 128.2, 52.0, 43.3, 40.2. HRMS (ESI, *m/z*): calculated for [M + Na] C_10_H_13_NO_2_Na, 202.0844, found 202.0840.

##### 4-(2-aminoethyl)benzamide **10b**

4-(2-aminoethyl)benzonitrile hydrochloride (500 mg, 1 eq) was solved in a mixture of EtOH/H_2_O (10 mL, 8:2 v/v) then KOH (1.63 g, 5 eq) was added. The solution was heated at 80 °C for 2 h. Water was added (5 mL), then a solution of Na_2_CO_3_ (10^−2^ M) until it reached pH=10. The solution was evaporated to dryness. The product was purified by chromatography on silica gel (DCM/5% NH_3_ in MeOH 6:4) to give 240 mg (53% yield) of orange/brown paste. m.p. 175 °C; ν_max_: 3352, 3237, 3125, 3055, 1661, 1559, 1383, 1054, 621 cm^−1^; ^1^H NMR (400 MHz, Methanol-*d*_4_) *δ* 7.89 (d, *^3^J* = 8.3 Hz, 2H), 7.41(d, *^3^J* = 8.3 Hz, 2H), 3.23 (m, 2H), 3.09 (m, 2H). ^13^C NMR (101 MHz, Methanol-*d*_4_) *δ* 177.1, 140.9, 132.5, 128.6, 127.9, 40.1, 33.0. HRMS (ESI, *m/z*): calculated for [M + H] C_9_H_13_N_2_O, 165.1028, found 165.1028.

#### 3.2.2. Synthesis of Compounds **4**

##### General Procedure

(*R*) or *(S*)-4-(oxiran-2-yl)-2,8-bis(trifluoromethyl)quinoline **9** (50 mg, 1 eq) was solved in ethanol (1mL), then the corresponding amine (3 eq) was added. The mixture was heated with a microwave oven at 130 °C and 150W for 30 min. The crude product was purified by chromatography on silica gel.

##### (*R*)-1-(2,8-bis(trifluoromethyl)quinolin-4-yl)-2-(phenethylamino)ethan-1-ol (**4a**)

(*R*)-4-(oxiran-2-yl)-2,8-bis(trifluoromethyl)quinoline was reacted with 2-phenylethan-1-amine (0.062 mL, 3 eq) according to the general procedure. The crude product was purified by chromatography on silica gel (DCM/5% NH_3_ in MeOH: 99/1) to give 51 mg (73% yield) of white solid. m.p. 160 °C; ν_max_: 3280, 2983, 2705, 2589, 2168, 1991, 1607, 1315, 1103, 1024, 866, 769, 703, 664 cm^−1^; ${\left[\alpha \right]}_{D}^{26}$ = −52.9 (*c* 0.1, DCM); ee = 99% (column Chiralpak IB, heptane/*i*PrOH 97:3, 1 mL/min, t_R_(*R*) = 15.7 min, t_R_(*S*) = 21.1 min); HRMS (ESI, *m/z*): calculated for [M + H] C_21_H_19_N_2_OF_6_, 429.1402, found 429.1398; ^1^H NMR (400 MHz, Chloroform-*d*) *δ* 8.18 (d, ^3^*J* = 8.9 Hz, 1H), 8.16 (d, ^3^*J* = 8.9 Hz, 1H), 8.09 (s, 1H), 7.71 (t, ^3^*J* = 8.0 Hz, 1H), 7.36–7.14 (m, 6H), 5.44 (dd, ^3^*J* = 8.8 Hz, ^3^*J* = 3.6 Hz, 1H), 3.17 (dd, ^2^*J* = 12.5 Hz, ^3^*J* = 3.7 Hz, 1H), 3.09–2.86 (m, 2H), 2.86–2.76 (m, 2H), 2.71 (dd, ^2^*J* = 12.5 Hz, ^3^*J* = 8.9 Hz, 1H). ^13^C NMR (101 MHz, Chloroform-*d*) *δ* 151.3, 143.7, 139.3, 128.7 (*^3^J_C-F_* = 4.1 Hz), 127.0, 126.9, 126.5, 114.5, 67.3, 55.4, 50.5, 36.5.

##### (*S*)-1-(2,8-bis(trifluoromethyl)quinolin-4-yl)-2-(phenethylamino)ethan-1-ol (**4b**)

(*S*)-4-(oxiran-2-yl)-2,8-bis(trifluoromethyl)quinoline was reacted with 2-phenylethan-1-amine (0.062 mL, 3 eq) according to the general procedure. The crude product was purified by chromatography on silica gel (DCM/5% NH_3_ in MeOH: 99/1) to give 44 mg (63% yield) of white solid. ${\left[\alpha \right]}_{D}^{26}$ = +53.0 (*c* 0.1, DCM); ee = 94% (column Chiralpak IB, heptane/*i*PrOH: 97/3, 1 mL/min, t_R_(*R*) = 15.8 min, t_R_(*S*) = 19.6 min); HRMS (ESI, *m/z*): calculated for [M + H] C_21_H_19_N_2_OF_6_, 429.1402, found 429.1399. NMR, IR spectra and mp were the same as **4a**.

##### (*R*)-1-(2,8-bis(trifluoromethyl)quinolin-4-yl)-2-((4-methylphenethyl)amino)ethan-1-ol (**4c**)

(*R*)-4-(oxiran-2-yl)-2,8-bis(trifluoromethyl)quinoline was reacted with 2-(*p*-tolyl)ethan-1-amine (0.071 mL, 3 eq) according to the general procedure. The crude product was purified by chromatography on silica gel (DCM/5% NH_3_ in MeOH: 99/1) to give 26 mg (37% yield) of white solid. m.p. 153 °C; ν_max_: 2950, 2925, 2840, 1600, 1516, 1462, 1305, 1107, 1038, 887, 835, 809, 766 cm^−1^; ${\left[\alpha \right]}_{D}^{23}$ = −52.9 (c 0.1, DCM); ee = 94% (column Chiralpak IB, heptane/iPrOH: 97/3, 0.5 mL/min, t_R_(*R*) = 35.5 min, t_R_(*S*) = 43.3 min); HRMS (ESI, m/z): calculated for [M + H] C_22_H_21_N_2_OF_6_, 443.1558, found 443.1558; ^1^H NMR (400 MHz, Chloroform-d) δ 8.21 (d, *^3^J* = 8.0 Hz, 1H), 8.18 (d, *^3^J* = 8.0 Hz, 1H), 8.12 (s, 1H), 7.73 (t, *^3^J* = 7.9 Hz, 1H), 7.12 (q, *^3^J* = 8.0 Hz, 4H), 5.47 (dd, *^3^J* = 8.9 Hz, *^3^J* = 3.6 Hz, 1H), 3.20 (dd, *^2^J* = 12.5 Hz, *^3^J* = 3.8 Hz, 1H), 3.04 (m, 1H), 2.94 (m, 1H), 2.82 (td, *^3^J* = 6.7 Hz, *^2^J* = 2.3 Hz, 2H), 2.73 (dd, *^2^J* = 12.6, *^3^J* = 8.8 Hz, 1H), 2.35 (s, 3H). ^13^C NMR (101 MHz, Chloroform-d) δ 148.4 (*^2^J*_C-F_ = 35.4 Hz), 136.1, 136.1, 128.7 (*^3^J*_C-F_ = 5.5 Hz), 128.4, 127.0, 126.9, 123.5 (*^1^J*_C-F_ = 276.7 Hz), 121.3 (*^1^J*_C-F_ = 276.7 Hz), 114.5 (*^3^J*_C-F_ = 2.0 Hz), 67.4, 55.4, 50.6, 35.9, 21.0.

##### (*S*)-1-(2,8-bis(trifluoromethyl)quinolin-4-yl)-2-((4-methylphenethyl)amino)ethan-1-ol (**4d**)

(S)-4-(oxiran-2-yl)-2,8-bis(trifluoromethyl)quinoline was reacted with 2-(p-tolyl)ethan-1-amine (0.071 mL, 3 eq) according to the general procedure. The crude product was purified by chromatography on silica gel (DCM/5% NH_3_ in MeOH: 99/1) to give 56 mg (79% yield) of white solid. ${\left[\alpha \right]}_{D}^{23}$ = +64.7 (c 0.1, DCM); ee = 97.5% (column Chiralpak IB, heptane/iPrOH: 97/3, 0.5 mL/min, t_R_(R) = 37.2 min, t_R_(S) = 41.2 min); HRMS (ESI, m/z): calculated for [M + H] C_22_H_21_N_2_OF_6_, 443.1558, found 443.1564. NMR, IR spectra and mp were the same as 4c.

##### (*R*)-1-(2,8-bis(trifluoromethyl)quinolin-4-yl)-2-((4-methoxyphenethyl)amino)ethan-1-ol (**4e**)

(*R*)-4-(oxiran-2-yl)-2,8-bis(trifluoromethyl)quinoline was reacted with 2-(4 methoxyphenyl)ethan-1-amine (0.072 mL, 3 eq) according to general the procedure. The crude product was purified by chromatography on silica gel (DCM/5% NH_3_ in MeOH: 99/1) to give 56 mg (80% yield) of white solid. m.p. 154 °C; ν_max_: 2957, 2938, 2841, 1607, 1512, 1453, 1306, 1247, 1104, 1035, 889, 815, 772 cm^−1^; ${\left[\alpha \right]}_{D}^{23}$ = −61.3 (c 0.1, DCM); ee = 95% (column Chiralpak IB, heptane/iPrOH: 95/5, 0.5 mL/min, t_R_(*R*) = 41.1 min, t_R_(*S*) = 49.8 min); HRMS (ESI, m/z): calculated for [M + H] C_22_H_21_N_2_O_2_F_6_, 459.1507, found 459.1512; ^1^H NMR (400 MHz, Chloroform-d) δ 8.21 (d, *^3^J* = 8.0 Hz, 1H), 8.18 (d, *^3^J* = 7.2 Hz, 1H), 8.12 (s, 1H), 7.73 (t, *^3^J* = 8.0 Hz, 1H), 7.13 (m, *^3^J* = 6.4 Hz, 2H), 6.88 (d, *^3^J* = 8.8 Hz, 2H), 5.47 (dd, *^3^J* = 8.8 Hz, *^3^J* = 3.7 Hz, 1H), 3.82 (s, 3H), 3.19 (dd, *^2^J* = 12.5 Hz, *^3^J* = 3.8 Hz, 1H), 3.02 (m, 1H), 2.93 (m, 1H), 2.80 (td, *^3^J* = 6.7 Hz, *^2^J* = 2.6 Hz, 2H), 2.74 (dd, *^2^J* = 12.5, *^3^J* = 8.7 Hz, 1H). ^13^C NMR (101 MHz, Chloroform-d) δ 158.3, 129.4, 128.6 (*^3^J*_C-F_ = 5.1 Hz), 127.0, 126.9, 121.2 (*^1^J*_C-F_ = 274.2 Hz), 113.5 (*^1^J*_C-F_ = 274.2 Hz), 114.5, 114.1, 67.3, 55.4, 55.3, 50.6, 35.5.

##### (*S*)-1-(2,8-bis(trifluoromethyl)quinolin-4-yl)-2-((4-methoxyphenethyl)amino)ethan-1-ol (**4f**)

(*S*)-4-(oxiran-2-yl)-2,8-bis(trifluoromethyl)quinoline was reacted with 2-(4 methoxyphenyl)ethan-1-amine (0.072 mL, 3 eq) according to the general procedure. The crude product was purified by chromatography on silica gel (DCM/5% NH_3_ in MeOH: 99/1) to give 64 mg (87% yield) of white solid. ${\left[\alpha \right]}_{D}^{23}$ = +64.6 (c 0.1, DCM); ee = 96% (column Chiralpak IB, heptane/iPrOH: 95/5, 0.5 mL/min, t_R_(*R*) = 45.2 min, t_R_(*S*) = 48.1 min); HRMS (ESI, m/z): calculated for [M + H] C_22_H_21_N_2_O_2_F_6_, 459.1507, found 459.1518. NMR, IR spectra and mp were the same as 4e.

##### (*R*)-1-(2,8-bis(trifluoromethyl)quinolin-4-yl)-2-((4-chlorophenethyl)amino)ethan-1-ol (**4g**)

(*R*)-4-(oxiran-2-yl)-2,8-bis(trifluoromethyl)quinoline was reacted with 2-(4-chlorophenyl)ethan-1-amine (0.072 mL, 3 eq) according to the general procedure. The crude product was purified by chromatography on silica gel (DCM/5% NH_3_ in MeOH: 99/1) to give 51 mg (68% yield) of white solid. m.p. 162 °C; ν_max_: 2945, 2922, 2853, 1601, 1520, 1455, 1309, 1109, 1039, 891, 819, 764, 703 cm^−1^; ${\left[\alpha \right]}_{D}^{23}$ = −56.7 (*c* 0.1, DCM); ee = 97% (column Chiralpak IA, heptane/*i*PrOH: 9/1, 1mL/min, t_R_(*R*) = 9.9 min, t_R_(*S*) = 28.2 min); HRMS (ESI, *m/z*): calculated for [M + H] C_21_H_18_N_2_O_2_F_6_Cl, 463.1012, found 463.1019; ^1^H NMR (400 MHz, Chloroform-*d*) *δ* 8.21 (d, ^3^*J* = 8.5 Hz, 1H), 8.19 (d, ^3^*J* = 7.2 Hz, 1H), 8.12 (s, 1H), 7.74 (t, ^3^*J* = 8.0 Hz, 1H), 7.31 (m, ^3^*J* = 8.4 Hz, 2H), 7.16 (d, ^3^*J* = 8.4 Hz, 2H), 5.47 (dd, ^3^*J* = 8.8 Hz, ^3^*J* = 3.6 Hz, 1H), 3.20 (dd, ^2^*J* = 12.5 Hz, ^3^*J* = 3.7 Hz, 1H), 3.07–2.91 (m, 2H), 2.89–2.80 (m, 2H), 2.74 (dd, ^2^*J* = 12.5 Hz, ^3^*J* = 8.9 Hz, 2H). ^13^C NMR (101 MHz, Chloroform-*d*) *δ* 151.2, 143.7, 137.7, 132.3, 130.0, 128.8, 127.0, 126.8, 126.6, 114.5, 67.4, 55.4, 50.4, 35.9.

##### (*S*)-1-(2,8-bis(trifluoromethyl)quinolin-4-yl)-2-((4-chlorophenethyl)amino)ethan-1-ol (**4h**)

(*S*)-4-(oxiran-2-yl)-2,8-bis(trifluoromethyl)quinoline was reacted with 2-(4-chlorophenyl)ethan-1-amine (0.072 mL, 3 eq) according to the general procedure. The crude product was purified by chromatography on silica gel (DCM/5% NH_3_ in MeOH: 99/1) to give 63 mg (84% yield) of white solid. ${\left[\alpha \right]}_{D}^{23}$ = +53.4 (*c* 0.1, DCM); ee = 94% (column Chiralpak IA, heptane/*i*PrOH 9:1, 1mL/min, t_R_(*R*) = 10.3 min, t_R_(*S*) = 27.6 min); HRMS (ESI, *m/z*): calculated for [M + H] C_21_H_18_N_2_O_2_F_6_Cl, 463.1012, found 463.1029. NMR, IR spectra and mp were the same as **4g**.

##### (*R*)-1-(2,8-bis(trifluoromethyl)quinolin-4-yl)-2-((4-nitrophenethyl)amino)ethan-1-ol (**4i**)

2-(4-nitrophenyl)ethan-1-amine hydrochloride (100 mg, 3 eq) was quenched by NaOH 1M then extracted with ethyl acetate. The combined organic layers were dried and the resulting solid was reacted with (*R*)-4-(oxiran-2-yl)-2,8-bis(trifluoromethyl)quinoline (50 mg, 1 eq) according to the general procedure. The crude product was purified by chromatography on silica gel (DCM then DCM/5% NH_3_ in MeOH: 99/1) to give 60 mg (78% yield) of white solid. m.p. 180 °C; ν_max_: 3279, 2921, 2854, 2018, 1602, 1518, 1308, 1108, 930, 849, 772, 702 cm^−1^; ${\left[\alpha \right]}_{D}^{26}$ = −32.6 (*c* 0.1, DCM); ee = 98% (column Chiralpak IA, heptane/*i*PrOH 9:1, 1 mL/min, t_R_(*R*) = 21.0 min, t_R_(*S*) = 53.3 min); HRMS (ESI, *m/z*): calculated for [M + H] C_21_H_18_N_3_O_3_F_6_, 474.1252, found 474.1260; ^1^H NMR (400 MHz, DMSO-*d*_6_) *δ* 8.63 (d, ^3^*J* = 8.8 Hz, 1H), 8.35 (d, ^3^*J* = 7.2 Hz, 1H), 8.10 (s, 1H), 8.06 (d, ^3^*J* = 8.8 Hz, 2H), 7.91 (t, ^3^*J* = 8.0 Hz, 1H), 7.39 (d, ^3^*J* = 8.4 Hz, 2H), 6.00 (d, ^3^*J* = 4.3 Hz, 1H), 5.58 (m, 1H), 2.94 (dd, ^3^*J* = 12.4, ^3^*J* = 4.3 Hz, 1H), 2.90–2.75 (m, 5H).^13^C NMR (101 MHz, DMSO-*d*_6_) *δ* 155.6, 149.5, 147.7 (*^2^J_C-F_* = 34.4 Hz), 146.2, 143.1, 130.3, 130.1 (*^3^J_C-F_* = 6.1 Hz), 129.8, 128.1, 123.6, 121.7 (*^1^J_C-F_* = 275.7 Hz), 115.3, 68.6, 56.6, 50.3, 36.0.

##### (*S*)-1-(2,8-bis(trifluoromethyl)quinolin-4-yl)-2-((4-nitrophenethyl)amino)ethan-1-ol (**4j**)

2-(4-nitrophenyl)ethan-1-amine hydrochloride (100 mg, 3 eq) was quenched by NaOH 1M then extracted with ethyl acetate. The combined organic layers were dried and the resulting solid was reacted with (*S*)-4-(oxiran-2-yl)-2,8-bis(trifluoromethyl)quinoline (50 mg, 1 eq) according to the general procedure. The crude product was purified by chromatography on silica gel (DCM then DCM/5% NH_3_ in MeOH: 99/1) to give 60 mg (78% yield) of white solid. ${\left[\alpha \right]}_{D}^{26}$ = +44.6 (*c* 0.1, DCM); ee = 94% (column Chiralpak IA, heptane/*i*PrOH: 9/1, 1 mL/min, t_R_(*R*) = 24.7 min, t_R_(*S*) = 57.7 min); HRMS (ESI, *m/z*): calculated for [M + H] C_21_H_18_N_3_O_3_F_6_, 474.1252, found 474.1251. NMR, IR spectra and mp were the same as **4i**.

##### Methyl (*R*)-4-(2-((2-(2,8-bis(trifluoromethyl)quinolin-4-yl)-2-hydroxyethyl)amino)ethyl)benzoate (**4k**)

(*R*)-4-(oxiran-2-yl)-2,8-bis(trifluoromethyl)quinoline was reacted with methyl 4-(2-aminoethyl)benzoate(90 mg, 3 eq) according to the general procedure. The crude product was purified by chromatography on silica gel (DCM/5% NH_3_ in MeOH: 99.5/0.5) to give 61 mg (77% yield) of white solid. m.p. 167 °C; ν_max_: 2952, 2918, 2847, 1715, 1607, 1542, 1434, 1282, 1105, 890, 768, 703 cm^−1^; ${\left[\alpha \right]}_{D}^{26}$ = −67.9 (*c* 0.1, DCM); ee = 99% (column Chiralpak IG, heptane/*i*PrOH: 9/1, 1mL/min, t_R_(*R*) = 11.5 min, t_R_(*S*) = 32.3 min); HRMS (ESI, *m/z*): calculated for [M + H] C_23_H_21_N_2_O_3_F_6_, 487.1456, found 487.1459; ^1^H NMR (400 MHz, Chloroform-*d*) *δ* 8.18 (d, ^3^*J* = 8.6 Hz, 1H), 8.16 (d, ^3^*J* = 7.4 Hz, 1H), 8.09 (s, 1H), 7.90 (d, ^3^*J* = 8.3 Hz, 2H), 7.71 (t, ^3^*J* = 7.9 Hz, 1H), 7.28 (d, ^3^*J* = 8.2 Hz, 2H), 5.45 (dd, ^3^*J* = 8.8 Hz,^3^*J* = 3.5 Hz, 1H), 3.92 (s, 3H), 3.18 (dd, ^2^*J* = 12.5 Hz, ^3^*J* = 3.7 Hz, 1H), 3.11–2.93 (m, 2H), 2.91–2.85 (m, 2H), 2.73 (dd, ^2^*J* = 12.6 Hz, ^3^*J* = 8.8 Hz, 1H).^13^C NMR (101 MHz, Chloroform-*d*) *δ* 166.9, 148.8 (*^2^J*_C-F_ = 35.7 Hz), 144.7, 130.0, 128.7, 128.7, 127.1, 126.8, 126.5, 114.5, 67.4, 55.4, 52.1, 50.2, 36.6.

##### Methyl (*S*)-4-(2-((2-(2,8-bis(trifluoromethyl)quinolin-4-yl)-2-hydroxyethyl)amino)ethyl)benzoate (**4l**)

(*S*)-4-(oxiran-2-yl)-2,8-bis(trifluoromethyl)quinoline was reacted with methyl 4-(2-aminoethyl)benzoate(90 mg, 3 eq) according to the general procedure. The crude product was purified by chromatography on silica gel (Et_2_O then Et_2_O/5% NH_3_ in MeOH: 99/1 to 97/3) to give 42 mg (53% yield) of white solid. ${\left[\alpha \right]}_{D}^{26}$ = +55.4 (*c* 0.1, DCM); ee = 94% (column Chiralpak IG, heptane/*i*PrOH: 9/1, 1mL/min, t_R_(*R*) = 11.6 min, t_R_(*S*) = 32.1 min); HRMS (ESI, *m/z*): calculated for [M + H] C_23_H_21_N_2_O_3_F_6_, 487.1456, found 487.1463. NMR, IR spectra and mp were the same as **4k**.

##### (*R*)-4-(2-((2-(2,8-bis(trifluoromethyl)quinolin-4-yl)-2-hydroxyethyl)amino)ethyl)benzonitrile (**4m**)

4-(2-aminoethyl)benzonitrile hydrochloride (90 mg, 3 eq) was quenched by NaOH 1M then extracted with ethyl acetate. The combined organic layers were dried and the resulting solid was reacted with (*R*)-4-(oxiran-2-yl)-2,8-bis(trifluoromethyl)quinoline (50 mg, 1 eq) according to the general procedure. The crude product was purified by chromatography on silica gel (Et_2_O then Et_2_O/5% NH_3_ in MeOH: 96.5/3.5) to give 64 mg (86% yield) of white solid. m.p. 140 °C; ν_max_: 2855, 2233, 1605, 1457, 1308, 1110, 988, 889, 830, 763, 703 cm^−1^; ${\left[\alpha \right]}_{D}^{26}$ = −63.6 (*c* 0.1, DCM); ee = 99% (column Chiralpak IG, heptane/*i*PrOH: 9/1, 1mL/min, t_R_(*R*) = 13.8 min, t_R_(*S*) = 39.8 min); HRMS (ESI, *m/z*): calculated for [M + H] C_22_H_18_N_3_OF_6_, 454.1354, found 454.1350; ^1^H NMR (400 MHz, Chloroform-*d*) *δ* 8.21 (d, ^3^*J* = 8.5 Hz, 1H), 8.19 (d, ^3^*J* = 8.5 Hz, 1H), 8.11 (s, 1H), 7.74 (t, ^3^*J* = 7.9 Hz 1H), 7.63 (d, ^3^*J* = 8.2 Hz, 2H), 7.34 (m, 2H), 5.49 (dd, ^3^*J* = 3.6 Hz, ^3^*J* = 8.8 Hz,1H), 3.21 (dd, ^2^*J* = 12.6 Hz, ^3^*J* = 3.7 Hz, 1H), 3.10–2.96 (m, 2H), 2.94–2.90 (m, 2H), 2.78 (dd, ^2^*J* = 12.6 Hz, ^3^*J* = 8.7 Hz, 1H).^13^C NMR (101 MHz, Chloroform-*d*) *δ* 151.0, 148.8 (*^2^J_C-F_*= 35.4 Hz), 150.0, 143.7, 132.4, 129.5, 128.8 (*^3^J_C-F_* = 5.8 Hz), 127.1, 126.8, 123.5 (*^1^J_C-F_* = 274.7 Hz), 121.1 (*^1^J_C-F_* = 276.7 Hz), 119.9, 114.5 (*^3^J_C-F_* = 2.0 Hz), 67.5, 55.4, 50.0, 36.7.

##### (*S*)-4-(2-((2-(2,8-bis(trifluoromethyl)quinolin-4-yl)-2-hydroxyethyl)amino)ethyl)benzonitrile (**4n**)

4-(2-aminoethyl)benzonitrile hydrochloride (90 mg, 3 eq) was quenched by NaOH 1M then extracted with ethyl acetate. The combined organic layers were dried and the resulting solid was reacted with (*S*)-4-(oxiran-2-yl)-2,8-bis(trifluoromethyl)quinoline (50 mg, 1 eq) according to the general procedure. The crude product was purified by chromatography on silica gel (Et_2_O then Et_2_O/5% NH_3_ in MeOH: 96/4) to give 62 mg (84% yield) of white solid. ${\left[\alpha \right]}_{D}^{26}$ = +45.1 (*c* 0.1, DCM); ee = 90% (column Chiralpak IG, heptane/*i*PrOH: 9/1, 1mL/min, t_R_(*R*) = 13.9 min, t_R_(*S*) = 39.2 min); HRMS (ESI, *m/z*): calculated for [M + H] C_22_H_18_N_3_OF_6_, 454.1354, found 454.1353. NMR, IR spectra and mp were the same as **4m**.

##### (*R*)-4-(2-((2-(2,8-bis(trifluoromethyl)quinolin-4-yl)-2-hydroxyethyl)amino)ethyl)benzoic acid (**4o**)

Methyl (*R*)-4-(2-((2-(2,8-bis(trifluoromethyl)quinolin-4-yl)-2-hydroxyethyl)amino)ethyl)benzoate **4k** (560 mg, 1 eq) was solved in THF/H_2_O (2 mL, *v/v*: 1/1), then LiOH.H_2_O (100 mg, 2 eq) was added. The mixture was heated at 68 °C during 16H. The crude product was quenched by HCl until pH=7. The precipitate formed during neutralization was filtered and washed with water. Thirty-nine milligrams (72 % yield) of a white solid were obtained. m.p. 217 °C; ν_max_: 3392, 2925, 1592, 1546, 1372, 1310, 1104, 767 cm^−1^; ${\left[\alpha \right]}_{D}^{26}$ = −26.1 (*c* 0.1, MeOH); HRMS (ESI, *m/z*): calculated for [M + H] C_22_H_19_N_2_O_3_F_6_, 473.1300, found 473.1295; ^1^H NMR (400 MHz, Methanol-*d*_4_) *δ* 8.46 (d, ^3^*J* = 8.5 Hz, 1H), 8.19 (d, ^3^*J* = 7.2 Hz, 1H), 8.11 (s, 1H), 7.82 (d, ^3^*J* = 8.0 Hz, 2H), 7.81 (t, ^3^*J* = 8.4 Hz, 1H), 7.21 (d, ^3^*J* = 8.0 Hz, 2H), 5.78 (dd, ^3^*J* = 2.9 Hz, ^3^*J* = 9.7 Hz, 1H), 3.29 (dd, ^2^*J* = 12.8 Hz, ^3^*J* = 2.8 Hz, 1H), 3.20–3.12 (m, 2H), 3.07 (dd, ^2^*J* = 12.8 Hz, ^3^*J* = 9.7 Hz, 1H), 2.96 (t, *^3^J_H16/H15_* = 7.8 Hz, 2H). ^13^C NMR (101 MHz, Methanol-*d*_4_) *δ* 150.9, 147.9, 140.6, 129.7, 129.0, 128.2, 128.0, 127.7, 114.6, 65.3, 52.9, 48.5, 31.9.

##### (*S*)-4-(2-((2-(2,8-bis(trifluoromethyl)quinolin-4-yl)-2-hydroxyethyl)amino)ethyl)benzoic acid (**4p**)

Methyl (*S*)-4-(2-((2-(2,8-bis(trifluoromethyl)quinolin-4-yl)-2 hydroxyethyl)amino)ethyl)benzoate **4l** (560 mg, 1 eq) was solved in THF/H_2_O (2 mL, 1:1 v/v), then LiOH.H_2_O (100 mg, 2 eq) was added. The mix was heated at 68 °C during 16H.The crude product was quenched by HCl until pH=7. The precipitate formed during neutralization was filtered and washed with water. Forty-five milligrams (68% yield) of a white solid were obtained. ${\left[\alpha \right]}_{D}^{26}$ = +43.6 (*c* 0.1, MeOH); HRMS (ESI, *m/z*): calculated for [M + H] C_22_H_19_N_2_O_3_F_6_, 454.1300, found 454.1297. NMR, IR spectra and mp were the same as **4o**.

##### (*R*)-4-(2-((2-(2,8-bis(trifluoromethyl)quinolin-4-yl)-2-hydroxyethyl)amino)ethyl)benzamide (**4q**)

(*R*)-4-(oxiran-2-yl)-2,8-bis(trifluoromethyl)quinoline was reacted with 4-(2-aminoethyl)benzamide (120 mg, 4.5 eq) according to the general procedure. The crude product was purified by chromatography on silica gel (DCM then DCM/5% NH_3_ in MeOH: 96/4) to give 20 mg (26% yield) of white solid. m.p. 155 °C; ν_max_: 3363, 3281, 3170, 2928, 2534, 1629, 1425, 1308, 1103, 764 cm^−1^; ${\left[\alpha \right]}_{D}^{26}$ = −37.7 (*c* 0.1, MeOH); HRMS (ESI, *m/z*): calculated for [M + H] C_22_H_20_N_3_O_2_F_6_, 472.1460, found 472.1466; ^1^H NMR (400 MHz, Methanol-*d*_4_) *δ* 8.39 (d, ^3^*J* = 8.6 Hz, 1H), 8.14 (d, ^3^*J* = 7.3 Hz, 1H), 8.02 (s, 1H), 7.75 (t, ^3^*J* = 8.0 Hz, 1H), 7.70 (d, ^3^*J* = 8.0 Hz, 2H), 7.21 (d, ^3^*J* = 8.0 Hz, 2H), 5.55 (dd, ^3^*J* = 3.0 Hz, ^3^*J* = 8.0 Hz, 1H), 2.93–2.88 (dd, ^2^*J* = 12.8 Hz, ^3^*J* = 3.2 Hz, 1H), 2.87–2.76 (m, 4H), 2.75 (dd, ^2^*J* = 12.4 Hz, ^3^*J* = 8.6 Hz, 1H). ^13^C NMR (101 MHz, Methanol-*d*_4_) δ 171.9, 153.3, 147.7 (*^2^J*_C-F_ = 34.4 Hz), 144.0, 143.4, 131.5, 128.8 (*^3^J*_C-F_ = 6.1 Hz), 128.5, 127.6, 127.5, 127.3, 123.8 (*^1^J*_C-F_ = 273.7 Hz), 121.1 (*^1^J*_C-F_ = 275.7 Hz), 114.2, 68.1, 55.8, 50.1, 35.3.

##### (*S*)-4-(2-((2-(2,8-bis(trifluoromethyl)quinolin-4-yl)-2-hydroxyethyl)amino)ethyl)benzamide (**4r**)

(*S*)-4-(oxiran-2-yl)-2,8-bis(trifluoromethyl)quinoline was reacted with 4-(2-aminoethyl)benzamide (120 mg, 4.5 eq) according to the general procedure. The crude product was purified by chromatography on silica gel (DCM then DCM/5% NH_3_ in MeOH: 96/4) to give 30 mg (39% yield) of white solid. ${\left[\alpha \right]}_{D}^{26}$ = +44.1 (*c* 0.1, MeOH); HRMS (ESI, *m/z*): calculated for [M + H] C_22_H_20_N_3_O_2_F_6_, 472.1460, found 472.1460. NMR, IR spectra and mp were the same as **4q**.

##### (*R*)-4-(2-((2-(2,8-bis(trifluoromethyl)quinolin-4-yl)-2-hydroxyethyl)amino)ethyl)benzene sulfonamide (**4s**)

(*R*)-4-(oxiran-2-yl)-2,8-bis(trifluoromethyl)quinoline was reacted with 4-(2-aminoethyl)benzenesulfonamide (98 mg, 3 eq) according to the general procedure. The crude product was purified by chromatography on silica gel (DCM then DCM/5% NH_3_ in MeOH: 97/3) to give 63 mg (76% yield) of white solid. m.p. 203 °C; ν_max_: 3419, 3304, 2932, 2856, 1596, 1311, 1158, 1100, 830, 691 cm^−1^; ${\left[\alpha \right]}_{D}^{26}$ = −40.0 (*c* 0.1, MeOH); HRMS (ESI, *m/z*): calculated for [M + H] C_21_H_20_N_3_O_3_F_6_S, 508.1130, found 508.1145; ^1^H NMR (400 MHz, Methanol-*d*_4_) *δ* 8.53 (d, ^3^*J* = 8.4 Hz, 1H), 8.28 (d, ^3^*J* = 7.1 Hz, 1H), 8.15 (s, 1H), 7.89 (t, ^3^*J* = 8.0 Hz, 1H), 7.85 (d, ^3^*J* = 8.3 Hz, 2H), 7.43 (d, ^3^*J* = 8.3 Hz, 2H), 5.68 (dd, ^3^*J* = 3.0 Hz, ^3^*J* = 8.8 Hz, 1H), 3.03 (dd, ^2^*J* = 12.4 Hz, ^3^*J* = 3.2 Hz, 1H), 3.00–2.90 (m, 4H), 2.86 (dd, ^2^*J* = 12.4 Hz, ^3^*J* = 8.8 Hz, 1H). ^13^C NMR (101 MHz, Methanol-*d*_4_) δ 144.5, 143.4, 141.6, 129.0, 128.9 (*^3^J*_C-F_ = 5.5 Hz), 127.7, 127.3, 126.6, 126.0, 121.1 (^1^J_C-F_ = 275.5 Hz), 123.8 (*^1^J*_C-F_ = 275.5 Hz), 114.2, 68.1, 55.8, 50.1, 35.3.

##### (*S*)-4-(2-((2-(2,8-bis(trifluoromethyl)quinolin-4-yl)-2-hydroxyethyl)amino)ethyl)benzene sulfonamide (**4t**)

(*S*)-4-(oxiran-2-yl)-2,8-bis(trifluoromethyl)quinoline was reacted with 4-(2-aminoethyl)benzenesulfonamide (98 mg, 3 eq) according to general procedure. The crude product was purified by chromatography on silica gel (DCM then DCM/5% NH_3_ in MeOH: 97/3) to give 64 mg (77% yield) of white solid. ${\left[\alpha \right]}_{D}^{26}$ = +43.0 (*c* 0.1, MeOH); HRMS (ESI, *m/z*): calculated for [M + H] C_21_H_20_N_3_O_3_F_6_S, 508.1130, found 508.1126. NMR, IR spectra and mp were the same as **4s**.

##### (*R*)-2-((4-aminophenethyl)amino)-1-(2,8-bis(trifluoromethyl)quinolin-4-yl)ethan-1-ol (**4u**)

(*R*)-4-(oxiran-2-yl)-2,8-bis(trifluoromethyl)quinoline was reacted with 4-(2-aminoethyl)aniline (68 µL, 3 eq) according to general procedure. The crude product was purified by chromatography on silica gel (DCM/5% solution NH_3_ in MeOH 99:1 then DCM/5% NH_3_ in MeOH: 97/3) to give 64 mg (89% yield) of white solid. m.p. 155 °C; ν_max_: 3286, 2928, 1606, 1514, 1433, 1372, 1305, 1101, 893, 829, 767, 702 cm^−1^; ${\left[\alpha \right]}_{D}^{26}$ = −40.9 (*c* 0.1, MeOH); HRMS (ESI, *m/z*): calculated for [M + H] C_21_H_20_N_3_OF_6_, 444.1511, found 444.1506; ^1^H NMR (400 MHz, Methanol-*d*_4_) *δ* 8.08 (d, ^3^*J* = 8.2 Hz, 1H), 8.06 (d, ^3^*J* = 7.3 Hz, 1H), 8.00 (s, 1H), 7.62 (t, ^3^*J* = 8.2 Hz, 1H), 6.87 (d, ^3^*J* = 8.3 Hz, 2H), 6.53 (d, ^3^*J* = 8.3 Hz, 2H), 5.36 (dd, ^3^*J* = 3.6 Hz, ^3^*J* = 8.7 Hz, 1H), 3.05 (dd, ^2^*J* = 12.5 Hz, ^3^*J* = 3.7 Hz, 1H), 2.86 (m, 2H), 2.65–2.58 (m, 2H). ^13^C NMR (101 MHz, Methanol-*d*_4_) δ 148.7 (*^2^J*_C-F_ = 35.1 Hz), 144.8, 143.5, 129.6, 129.0, 128.7 (*^3^J*_C-F_ = 5.4 Hz), 127.3, 127.0, 126.5, 123.3 (*^1^J*_C-F_ = 274.9 Hz), 120.9 (*^1^J*_C-F_ = 275.6 Hz), 115.4, 114.5, 67.4, 55.4, 50.7, 35.3.

##### (*S*)-2-((4-aminophenethyl)amino)-1-(2,8-bis(trifluoromethyl)quinolin-4-yl)ethan-1-ol (**4v**)

(*S*)-4-(oxiran-2-yl)-2,8-bis(trifluoromethyl)quinoline was reacted with 4-(2-aminoethyl)aniline (68 µL, 3 eq) according to the general procedure. The crude product was purified by chromatography on silica gel (DCM/5% solution NH_3_ in MeOH: 99/1 to 97/3) to give 64 mg (89% yield) of white solid. ${\left[\alpha \right]}_{D}^{26}$ = +53.4 (*c* 0.1, MeOH); HRMS (ESI, *m/z*): calculated for [M + H] C_21_H_20_N_3_OF_6_, 444.1511, found = 444.1508. NMR, IR spectra and mp were the same as **4u**.

### 3.3. Antibacterial Assays

The following strains were used for testing antibacterial susceptibility to **4a**–**4v** and **3m/3n**: *Escherichia coli* DSM 1103 and *Pseudomonas aeruginosa* DSM 1117 for Gram-negative bacteria (Deutsche Sammlung für Mikroorganismen, Braunschweig, Germany) and *Staphylococcus aureus* CIP 103.429 and *Enterococcus faecalis* CIP 103.214 (Collection de l’Institut Pasteur, Paris, France) for Gram-positive bacteria. Bacteria were grown overnight at 35 °C in Tryptic Soy Broth and streaked on Tryptic Soy Agar (TSA) (AES, Bruz, France). From these isolation plates, inocula were prepared according to CLSI recommendations [27] and the broth microdilution technique carried out in Mueller–Hinton Broth (pH 7.4) as advised using drug concentrations ranging from 0.0625 to 128 µg/mL obtained from serial two-fold dilutions of stock solutions of compounds 3 or 4 in DMSO (Sigma-Aldrich, Saint-Quentin Fallavier, France). Ciprofloxacin (Sigma-Aldrich, France) was used as control in each series of experiments. The minimum inhibitory concentration (MIC) was determined as the lowest concentration at which wells remained visually clear.

### 3.4. Antimycobacterial Tests

Susceptibility testing by broth microdilution technique was performed in duplicate on *Mycobacterium avium* ATCC 700,898 (also known as « MAC 101 ») according to CLSI guidelines [28]. Inocula were prepared in saline solution using transparent colonies and standardized using a nephelemeter, then diluted in the appropriate media to obtained a final solution around 5 × 10^5^ CFU/mL. As recommended by CLSI, the susceptibility testing was first realized in CAMHB medium (Sigma-Aldrich, France) complemented with 5% OADC (Becton, Dickinson and Company, Le-Pont-de-Claix, France). Also, the same procedure was applied in MB 7H9 medium (Sigma-Aldrich, Saint-Quentin Fallavier, France) + 5% OADC to avoid potential medium-dependent activity of the tested compounds. Ninety-six-wells transparent polystyrene microplates (Thermo-Scientific, Illkirch, France) were incubated static at 37 °C during 14 days before reading. A clarithromycin (Sigma-Aldrich, France) stock solution in acetone was used as control in each series of experiments and results were compared with CLSI breakpoints. Activities of the compounds synthetized during this study were determined for high drug concentrations ranging from 2 to 64 µg/mL. The MIC was determined as the lowest concentration at which wells remained visually clear.

## 4. Conclusions

We have prepared more than twenty novel quinoline-based drugs **4** in a five-step asymmetric synthesis with good enantiomeric excesses (>90%). These compounds were either active against Gram-positive bacteria (MIC ≤ 4 µg/mL for **4c**–**4h** and **4k**/**4l**) or *E. coli* (MIC = 32–64 µg/mL for **4q**–**4v**) according to the global lipophilicity of the compounds. Unfortunately, these quinolines **4** were weakly active against *M. avium*. Interestingly, all compounds **3** of the previously synthesized series were efficient on *M. avium* with MIC = 2–16 µg/mL, whatever the clogP value and the side chain length. This study has confirmed the strong antibacterial potential of quinoline-based drugs in relation with their lipophilicity. Further studies are under progress in order to better understand their mechanism(s) of action.

## Figures and Tables

**Table 1:** Antibacterial activities of compounds **3a**–**3n**.

Compound	MIC (µg/mL) ^a^				clogP ^b^
	*S. aureus* *CIP 103.429*	*E. faecalis* *CIP 103.214*	*E. coli* *DSM 1103*	*P. aeruginosa* *DSM 1117*	
**3a (3b)**	16 (16)	16 (16)	32 (64)	>128 (>128)	4.30
**3c (3d)**	16 (16)	16 (16)	16 (32)	>128 (>128)	4.59
**3e (3f)**	4 (4)	4 (4)	>128 (16)	>128 (>128)	4.95
**3g (3h)**	1 (2)	1 (2)	>128 (>128)	>128 (>128)	5.31
**3i (3j)**	1 (1)	1 (1)	>128 (>128)	>128 (>128)	5.70
**3k (3l)**	16 (>128)	16 (>128)	>128 (>128)	>128 (>128)	6.01
**3m (3n)**	8 (8)	8 (8)	8 (16)	>128 (>128)	4.46
**mefloquine**	16	32	64	>128	ND^c^
**ciprofloxacin**	0.25	0.25	0.0625	0.125	ND

^a^ Minimum inhibitory concentration. ^b^ logP calculated with Qikprop, a Schrödinger software. ^c^ Not determined.

**Table 2:** Yields, enantiomeric excesses and optical rotation of compounds **4**.

N°	R_2_	AC ^a^	Yield (%)	ee ^b^	$\alpha_{D}^{T}$ ^c^	N°	R_2_	AC ^a^	Yield (%)	ee ^b^	$\alpha_{D}^{T}$ ^c^
**4a**	H	*R*	73	99	−52.9	**4l**	COOMe	*S*	53	94	+55.4
**4b**		*S*	63	94	+53.0						
**4c**	Me	*R*	37	94	−52.9	**4m**	CN	*R*	86	99	−63.6
**4d**		*S*	79	97.5	+64.7	**4n**		*S*	84	90	+45.1
**4e**	OMe	*R*	80	95	−61.3	**4o**	COOH	*R*	72	ND ^d^	−26.1
**4f**		*S*	87	96	+64.6	**4p**		*S*	68	ND	+43.6
**4g**	Cl	*R*	68	97	−56.7	**4q**	CONH_2_	*R*	39	ND	−37.7
**4h**		*S*	84	94	+53.4	**4r**		*S*	26	ND	+44.1
**4i**	NO_2_	*R*	78	98	−32.6	**4s**	SO_2_NH_2_	*R*	76	ND	−40.0
**4j**		*S*	61	94	+44.6	**4t**		*S*	77	ND	+43.0
**4k**	COOMe	*R*	77	99	−67.9	**4u**	NH_2_	*R*	89	ND	−40.9
						**4v**		*S*	89	ND	+53.4

^a^ Absolute configuration. ^b^ Enantiomeric excess in % determined by HPLC, see conditions to experimental Section 3.2.2. ^c^ Optical rotation, see concentration, solvent and T to experimental Section 3.2.2. ^d^ Not determined.

**Table 3:** Antibacterial activities and physicochemical constant of compounds **4** vs. **3m**, **3n** and mefloquine (MQ).

N°	R_2_	AC ^a^	MIC (µg/mL) ^b^				Physicochemical Constant		
			*S. aureus* *CIP103.429*	*E. faecalis* *CIP 103214*	*E. coli* *DSM 1103*	*P. aeruginosa* *DSM 1117*	σ_p_^c^	π ^f^	clogP ^g^
**4a**	**H**	*R*	8	8	>128	>128	0	0	5.23
**4b**		*S*	8	8	>128	>128			
**4c**	Me	*R*	2	4	>128	>128	–0.170	0.56	5.47
**4d**		*S*	2	2	>128	>128			
**4e**	OMe	*R*	4	8	>128	32	−0.268	−0.02	5.12
**4f**		*S*	4	8	>128	>128			
**4g**	Cl	*R*	1	2	>128	>128	0.227	0.71	5.75
**4h**		*S*	1	2	>128	>128			
**4i**	NO_2_	*R*	64	128	>128	>128	0.778^d^	−0.28	4.49
**4j**		*S*	64	128	>128	>128			
**4k**	COOMe	*R*	4	8	>128	>128	0.619^e^	−0.01	4.37
**4l**		*S*	4	4	>128	>128			
**4m**	CN	*R*	8	>128	>128	>128	0.660	−0.57	4.52
**4n**		*S*	8	>128	>128	>128			
**4o**	COOH	*R*	64	64	>128	>128	0.257	−0.28	2.33
**4p**		*S*	64	64	>128	>128			
**4q**	CONH_2_	*R*	32	32	64	>128	0.627	−1.49	3.58
**4r**		*S*	16	32	64	>128			
**4s**	SO_2_NH_2_	*R*	16	32	64	>128	0.621	−1.82	2.90
**4t**		*S*	16	16	64	>128			
**4u**	NH_2_	*R*	8	8	32	>128	−0.660	−1.23	4.12
**4v**		*S*	8	16	64	>128			
**3m**	OH	*R*	8	8	8	>128	−0.357	−0.67	4.46
**3n**		*S*	8	8	16	>128			
**mefloquine**			16	32	64	>128			
**ciprofloxacin**			0.25	0.25	0.0625	0.125			

^a^ Absolute configuration. ^b^ Minimum inhibitory concentration. ^c^ Hammett’s para substituent constant [29]. ^d^ [30]. ^e^ Hammett’s para substituent constant—substituent constant for reactions of phenols and anilines. ^f^ Hydrophobic substituent value—values from benzene series used—[31]. ^g^ logP calculated with Qikprop, a Schrödinger software.

**Table 4:** Activity against *M. avium* for compounds of series **3** and **4**.

	Compound		MIC (µg/mL) ^b^		clogP ^e^
			*M. avium* ATCC 700898 in CAMBH ^c^	*M. avium* ATCC 700898 in MB 7H9 ^d^	
	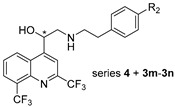				
**N°**	**R_2_**	**AC^a^**			
**4a**	**H**	*R*	>64	>64	5.23
**4b**		*S*	>64	>64	
**4c**	Me	*R*	>64	>64	5.47
**4d**		*S*	>64	>64	
**4e**	OMe	*R*	>64	>64	5.12
**4f**		*S*	>64	>64	
**4g**	Cl	*R*	>64	>64	5.75
**4h**		*S*	>64	>64	
**4i**	NO_2_	*R*	>64	>64	4.49
**4j**		*S*	>64	>64	
**4k**	COOMe	*R*	>64	>64	4.37
**4l**		*S*	>64	>64	
**4m**	CN	*R*	>64	>64	4.52
**4n**		*S*	>64	>64	
**4o**	COOH	*R*	>64	>64	2.33
**4p**		*S*	>64	>64	
**4q**	CONH_2_	*R*	64	64	3.58
**4r**		*S*	64	64	
**4s**	SO_2_NH_2_	*R*	64	64	2.90
**4t**		*S*	64	64	
**4u**	NH_2_	*R*	64	64	4.12
**4v**		*S*	64	64	
**3m**	OH	*R*	32	32	4.46
**3n**		*S*	32	32	
	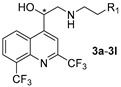				
	**R_1_**	**AC*^a^***			
**3a**	-CH_2_CH_3_	*R*	4	16	4.30
**3b**		*S*	8	32	
**3c**	-(CH_2_)_2_CH_3_	*R*	4	16	4.59
**3d**		*S*	8	8	
**3e**	-(CH_2_)_3_CH_3_	*R*	8–16 ^f^	8–16 ^f^	4.95
**3f**		*S*	8–16 ^f^	8–16 ^f^	
**3g**	-(CH_2_)_4_CH_3_	*R*	8	16	5.31
**3h**		*S*	4	4	
**3i**	-(CH_2_)_5_CH_3_	*R*	ND ^g^	ND	5.70
**3j**		*S*	4	4	
**3k**	-(CH_2_)_6_CH_3_	*R*	8–16 ^f^	8–16 ^f^	6.01
**3l**		*S*	2	8	
**mefloquine racemic**			4	16	ND
**mefloquine 1a**		*R,S*	4	16	
**mefloquine 1b**		*S,R*	4	8	
**bedaquiline 2a**			≤0.063	≤0.063	ND
**rifampin**			64	32–64	ND
**clarithromycin**			1	4–8	ND
**ethambutol**			8–16	8	ND
**ciprofloxacin**			8	4–8	ND

^a^ Absolute configuration. ^b^ Minimum inhibitory concentration. The experiments were carried out in duplicate. ^c^ culture medium = cation-adjusted Mueller-Hinton Broth (CAMHB) + 5% OADC(pH = 7.3). ^d^ culture medium = MiddleBrook 7H9 + 5% OADC (pH = 6.8). ^e^ logP calculated with Qikprop, a Schrödinger software. ^f^ Different values were obtained in experiment 1 and 2. ^g^ Not determined.

**Table 5:** Structure-antimicrobial activity relationships for compounds **3** and **4**.

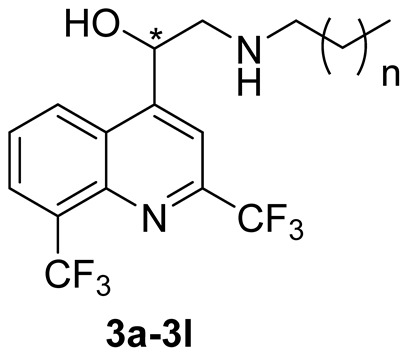	**clogP < 4.6**	**4.6 < clogP < 6**	**clogP > 6**
	n = 2–3 active against *E. coli* (MIC = 16–64 µg/mL).	n = 4–6 activity is increased against Gram-positive bacteria (MIC = 1–4 µg/mL).	n = 7 **3l** is the lead compound against *M. avium* (MIC = 2 µg/mL).
	All compounds were active against ***M. avium*** whatever the clogP value		
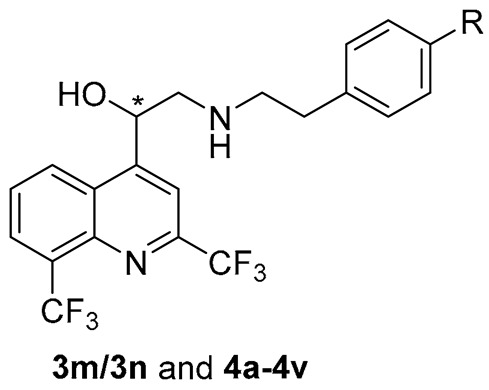	**clogP < 4**	**4 < clogP < 5**	**clogP > 5**
	R = COOH, CONH_2_, SO_2_NH_2_ activity is decreased against Gram-positive bacteria (MIC = 16–64 µg/mL) and weak on *E. coli* and *M. avium* (MIC = 64 µg/mL).	−σ and −π (R = NH_2_, OH): activity is increased against *E. coli* (MIC = 8–64 µg/mL) and *M. avium* (MIC =32–64 µg/mL) and retained against Gram-positive bacteria (MIC = 8–16 µg/mL). +σ and +π (R = NO_2_): activity is weak against Gram-positive bacteria and no activity against *E. coli.*	R = H, Me, OMe, Cl activity is increased against Gram-positive bacteria (MIC = 1–8 µg/mL).

## Supplementary Materials

All ^1^H and ^13^C NMR spectra of compounds **4a**–**4u** (Figures S1–S22) are available online at https://www.mdpi.com/1424-8247/12/2/91/s1.
