# Supplementary material for: Synthesis and Study of New Quinolineaminoethanols as Anti-Bacterial Drugs
Source: Pharmaceuticals (Basel). 2019 Jun 18;12(2):91. doi: 10.3390/ph12020091 (PMC6630482; doi:10.3390/ph12020091)

Supplementary Materials

Synthesis and Study of New Quinolineaminoethanols as Anti-Bacterial Drugs

Pierre Laumaillé[1], Alexandra Dassonville-Klimpt[1,*], François Peltier[1,2], Catherine Mullié[1], Claire Andréjak[1,3], Sophie Da-Nascimento[1], Sandrine Castelain[1,2], Pascal Sonnet[1,*]

[1] AGIR, EA 4294, UFR of Pharmacy, Jules Verne University of Picardie, 80037, Amiens, France
[2] Department of Bacteriology, Amiens University Hospital, Amiens, France.
[3] Respiratory and Intensive Care Unit, University Hospital Amiens, Amiens, 80054, France.
* Correspondence: alexandra.dassonville@u-picardie.fr and pascal.sonnet@u-picardie.fr

NMR spectra of compounds **4a**-**4v**

Figure S1: ^{1}H NMR of compound **4a** (400 MHz, Chloroform-*d*)

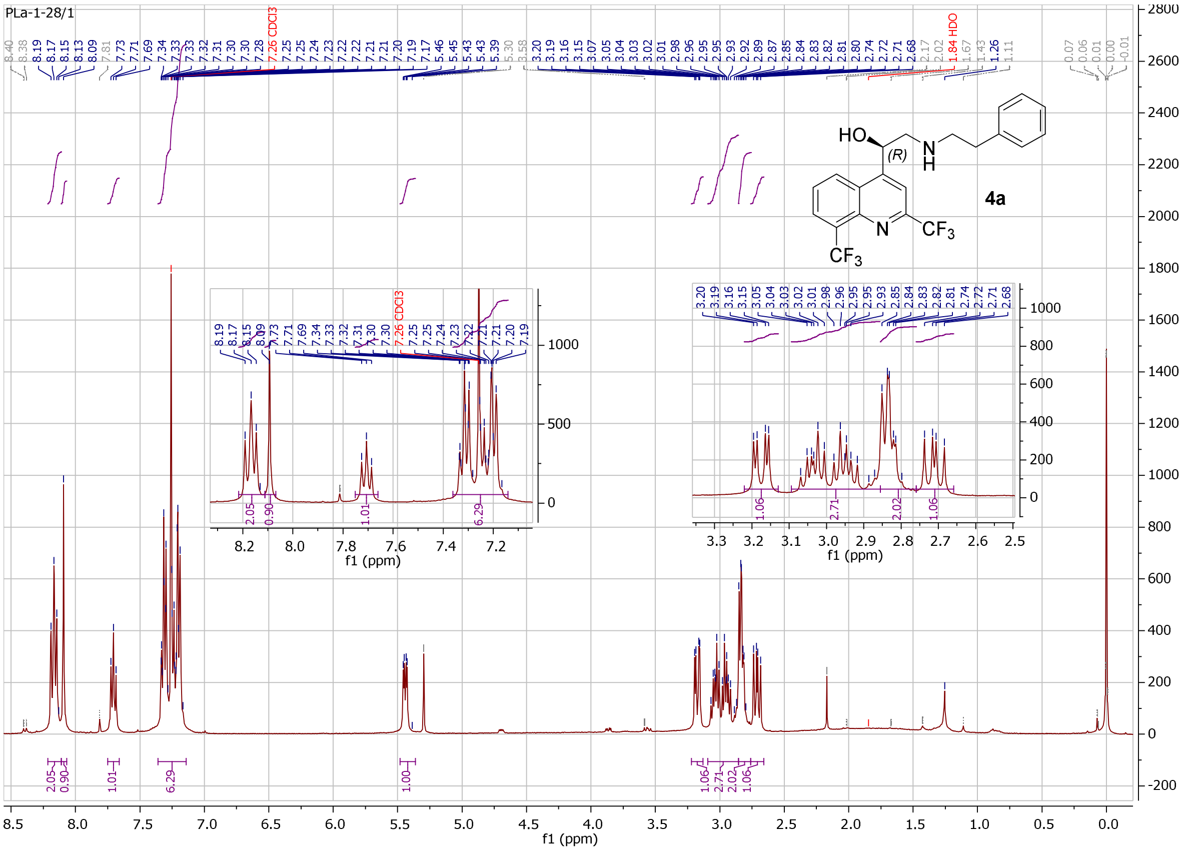

Figure S2: ^{13}C NMR of compound **4a** (101 MHz, Chloroform-*d*)

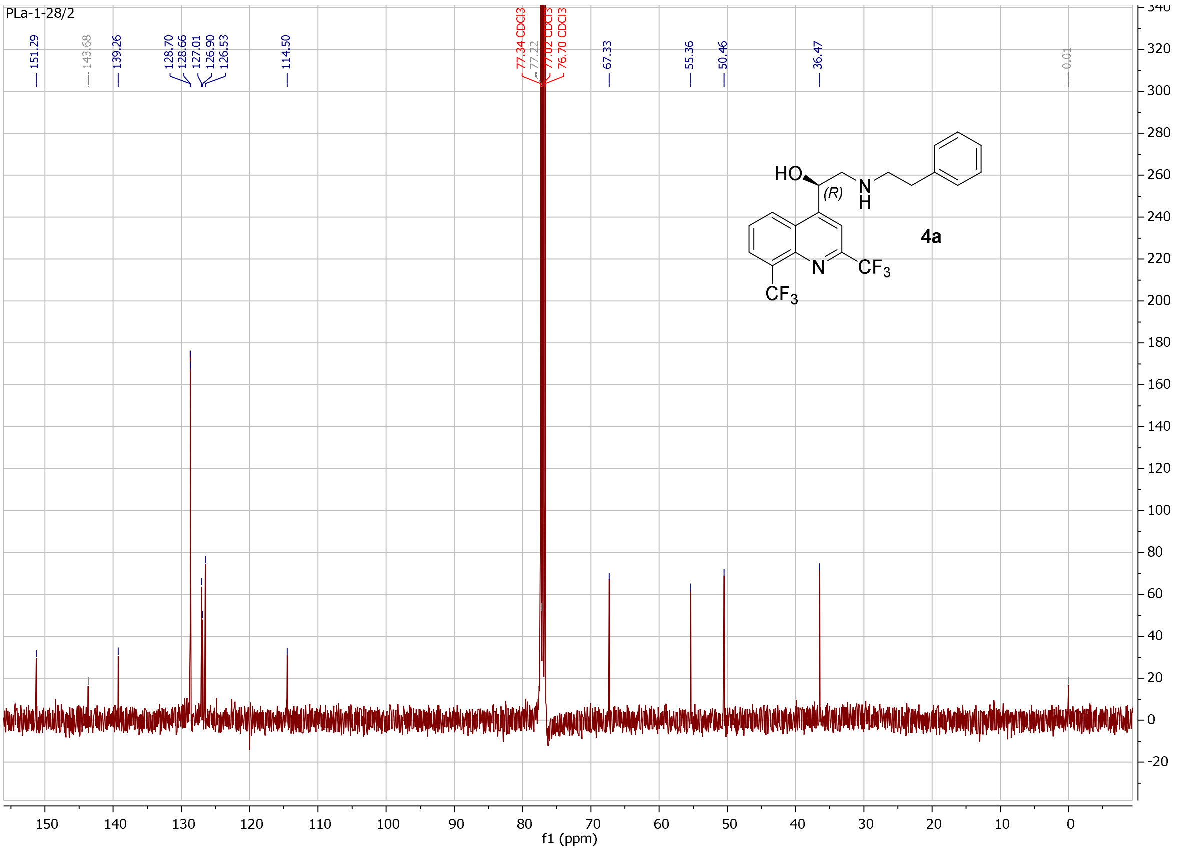

Figure S3: ^{1}H NMR of compound **4c** (400 MHz, Chloroform-*d*)

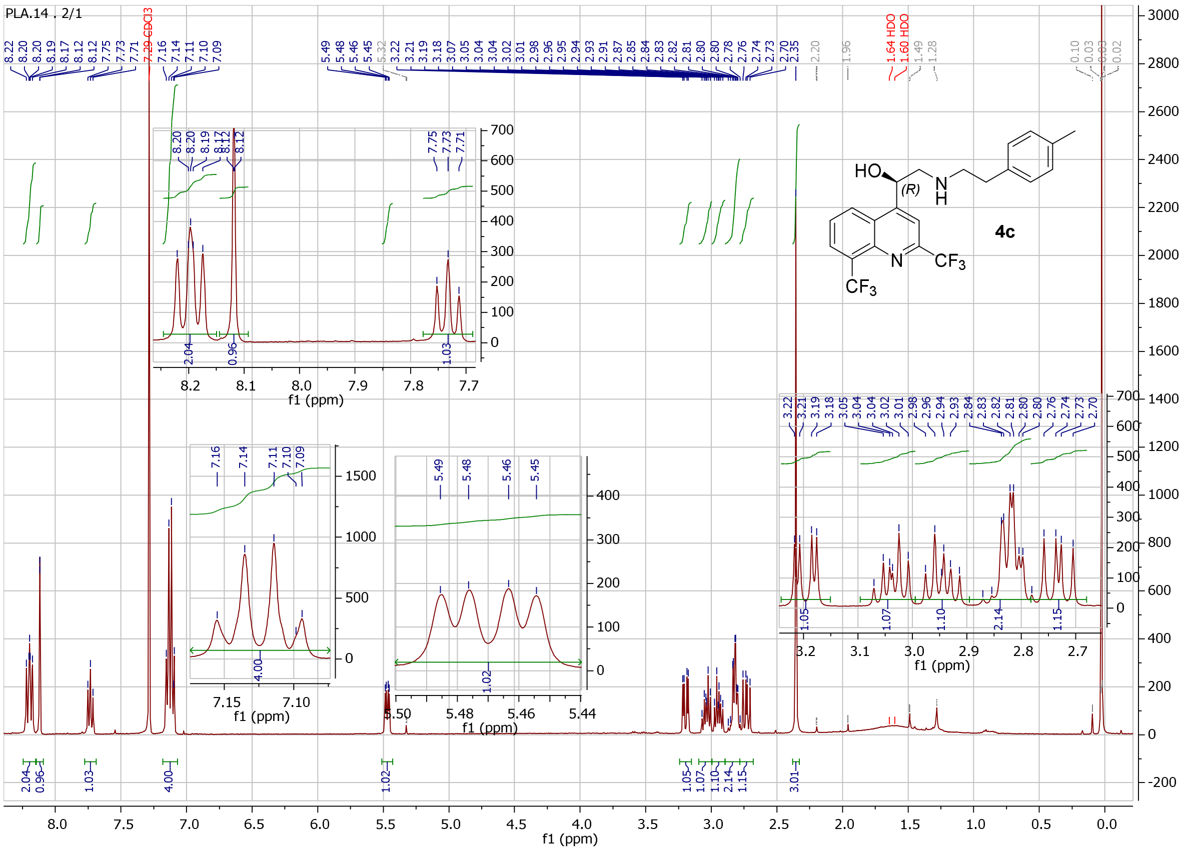

Figure S4: ^{13}C NMR of compound **4c** (101 MHz, Chloroform-*d*)

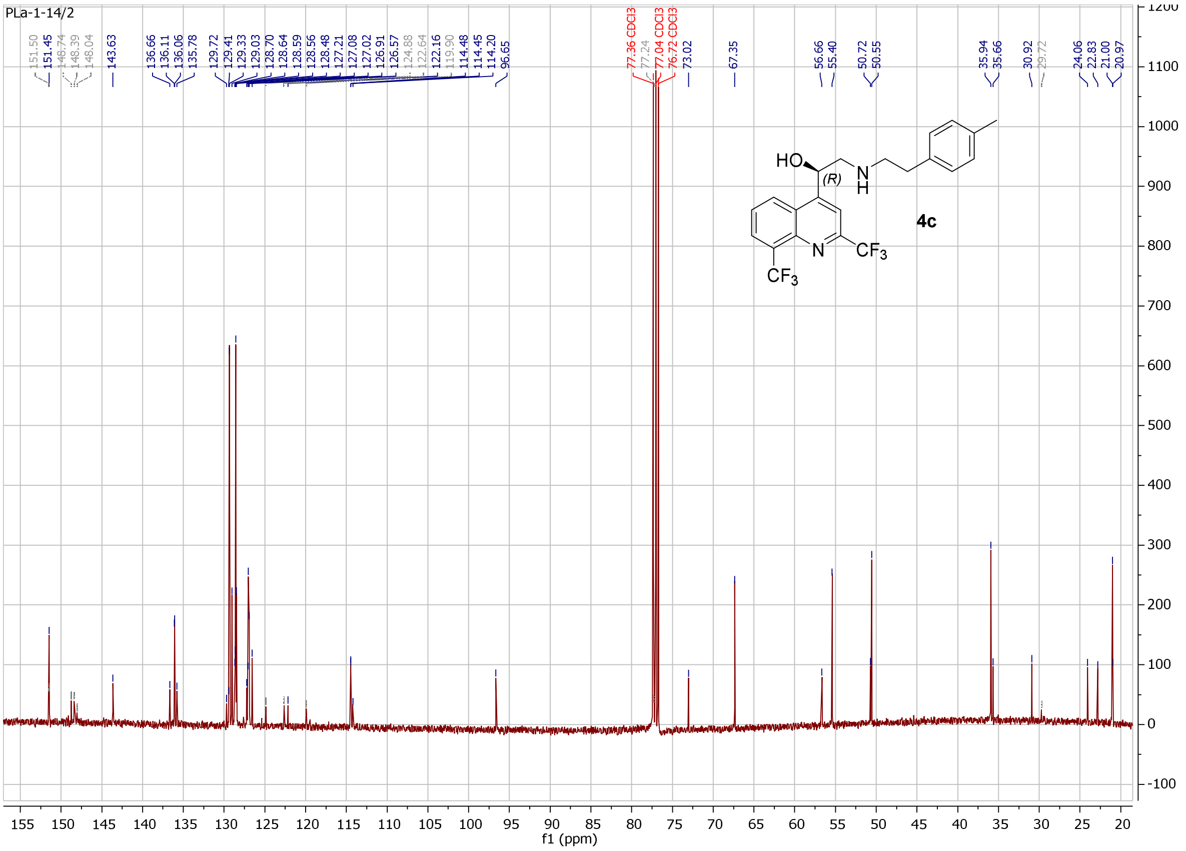

Figure S5: ^{1}H NMR of compound **4e** (400 MHz, Chloroform-*d*)

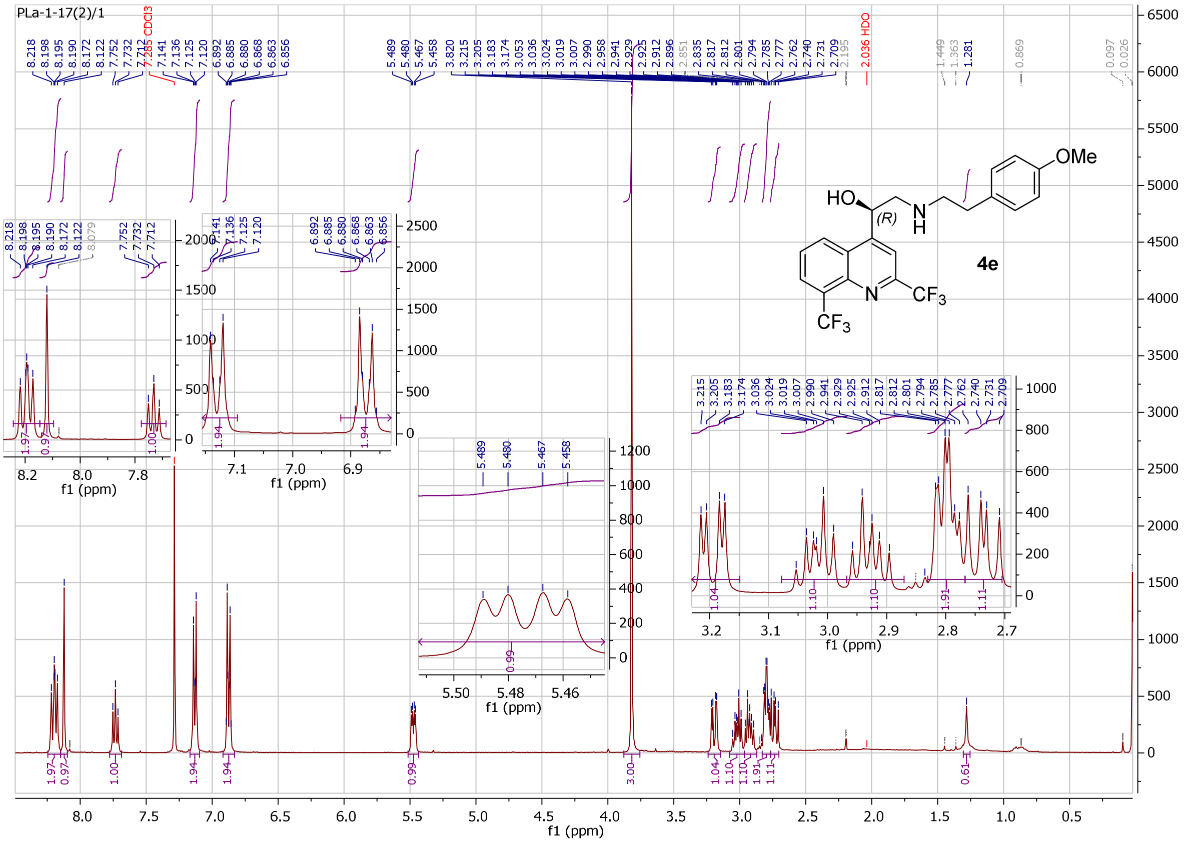

Figure S6: ^{13}C NMR of compound **4e** (101 MHz, Chloroform-*d*)

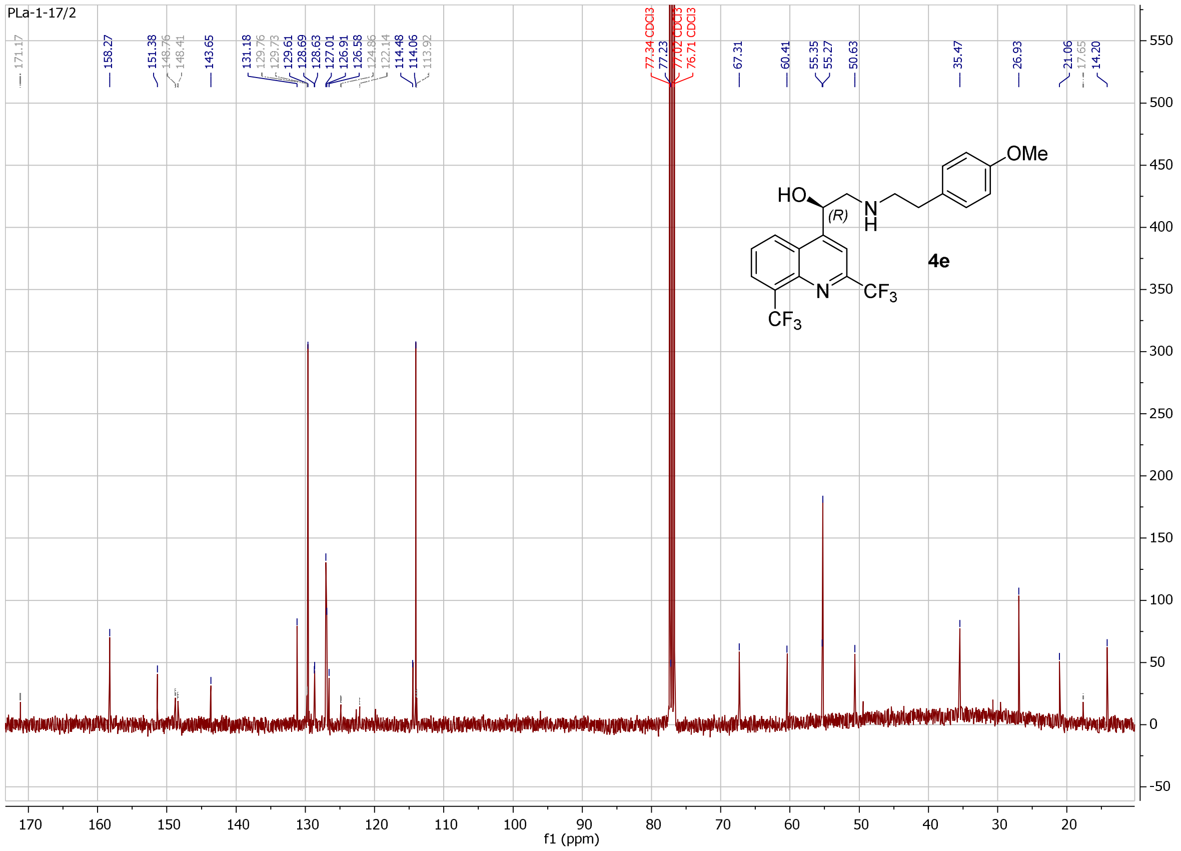

Figure S7: ^{1}H NMR of compound **4g** (400 MHz, Chloroform-*d*)

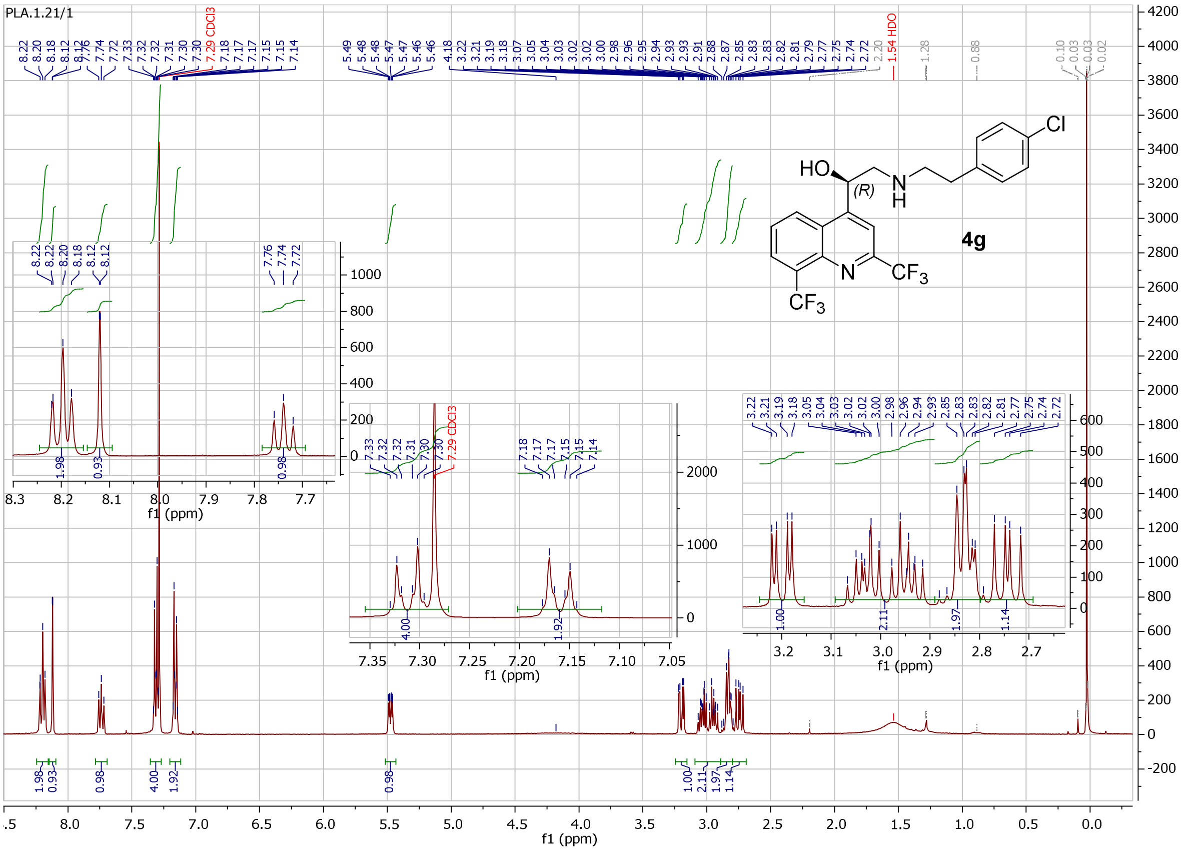

Figure S8: ^{13}C NMR of compound **4g** (101 MHz, Chloroform-*d*)

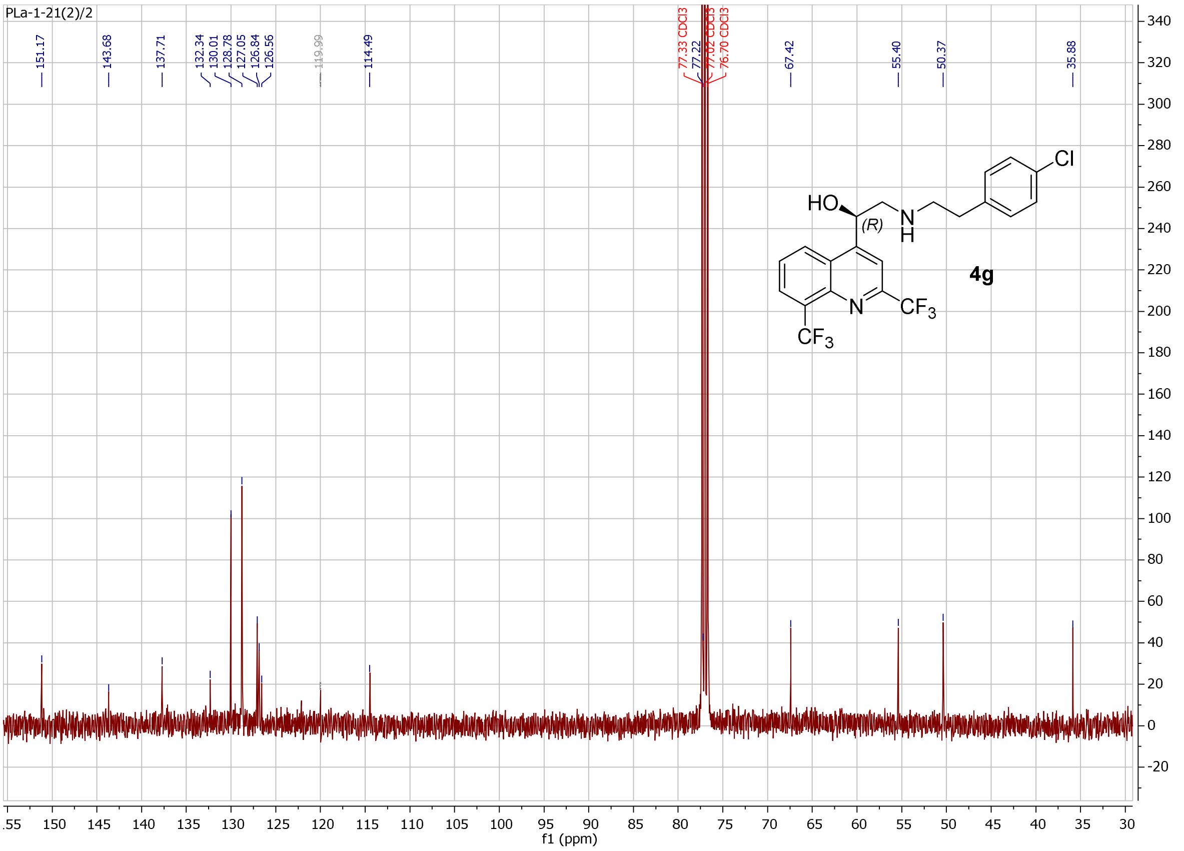

Figure S9: ^{1}H NMR of compound **4i** (400 MHz, DMSO-d_6)

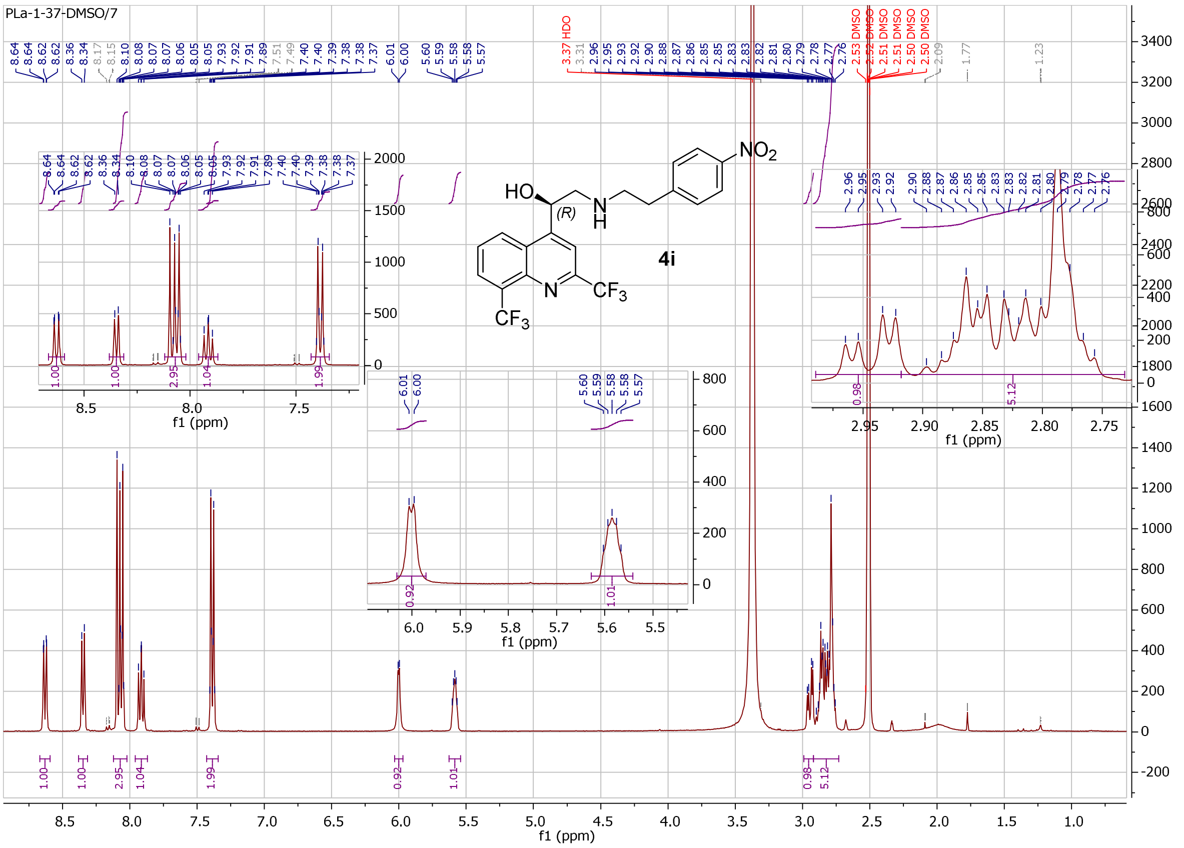

Figure S10: ^{13}C NMR of compound **4i** (101 MHz, DMSO-d_6)

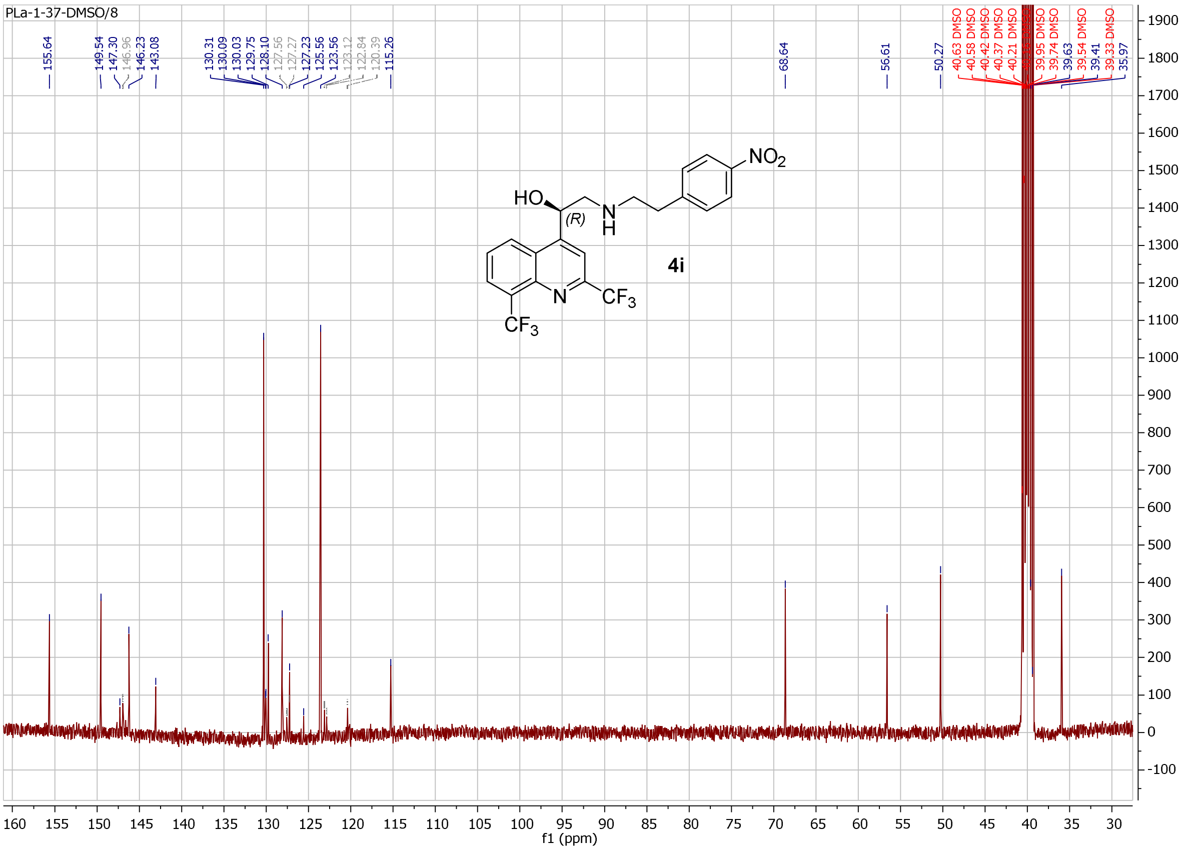

Figure S11: ^{1}H NMR of compound **4k** (400 MHz, Chloroform-*d*)

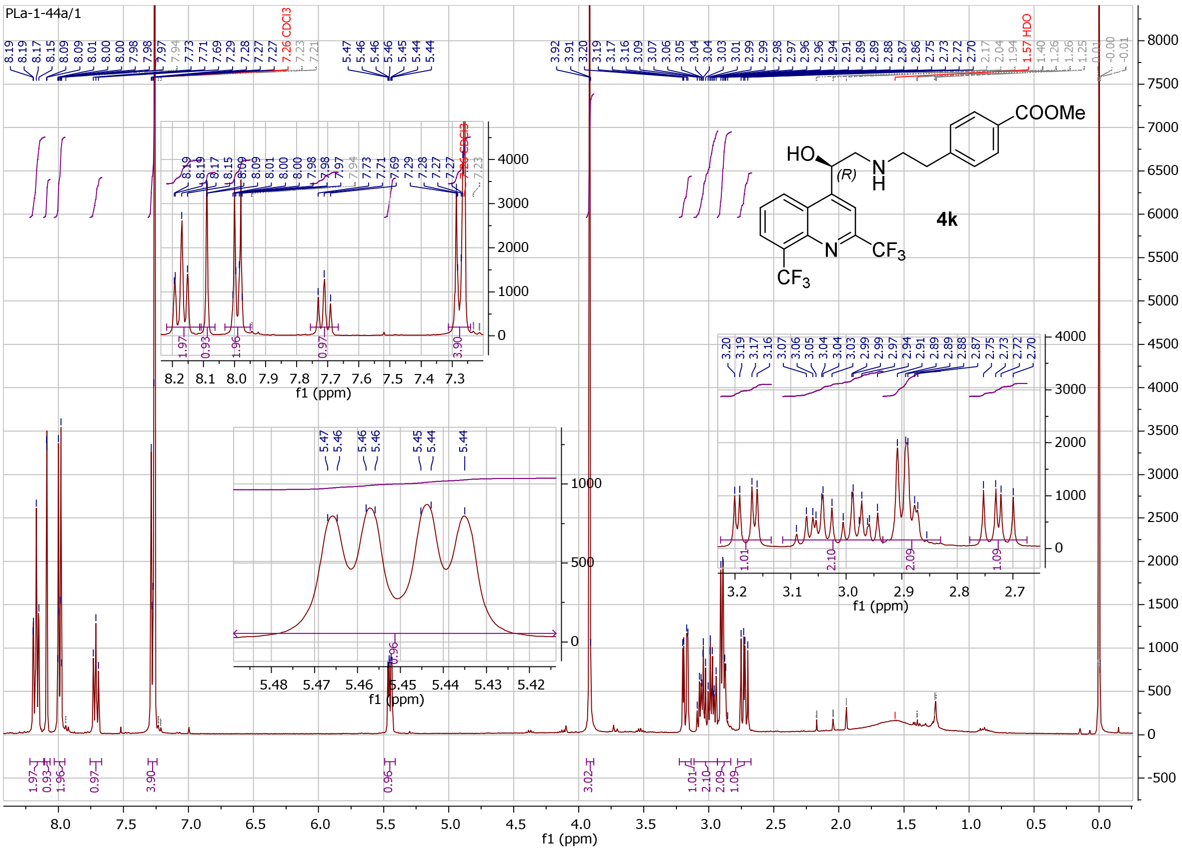

Figure S12: ^{13}C NMR of compound **4k** (101 MHz, Chloroform-*d*)

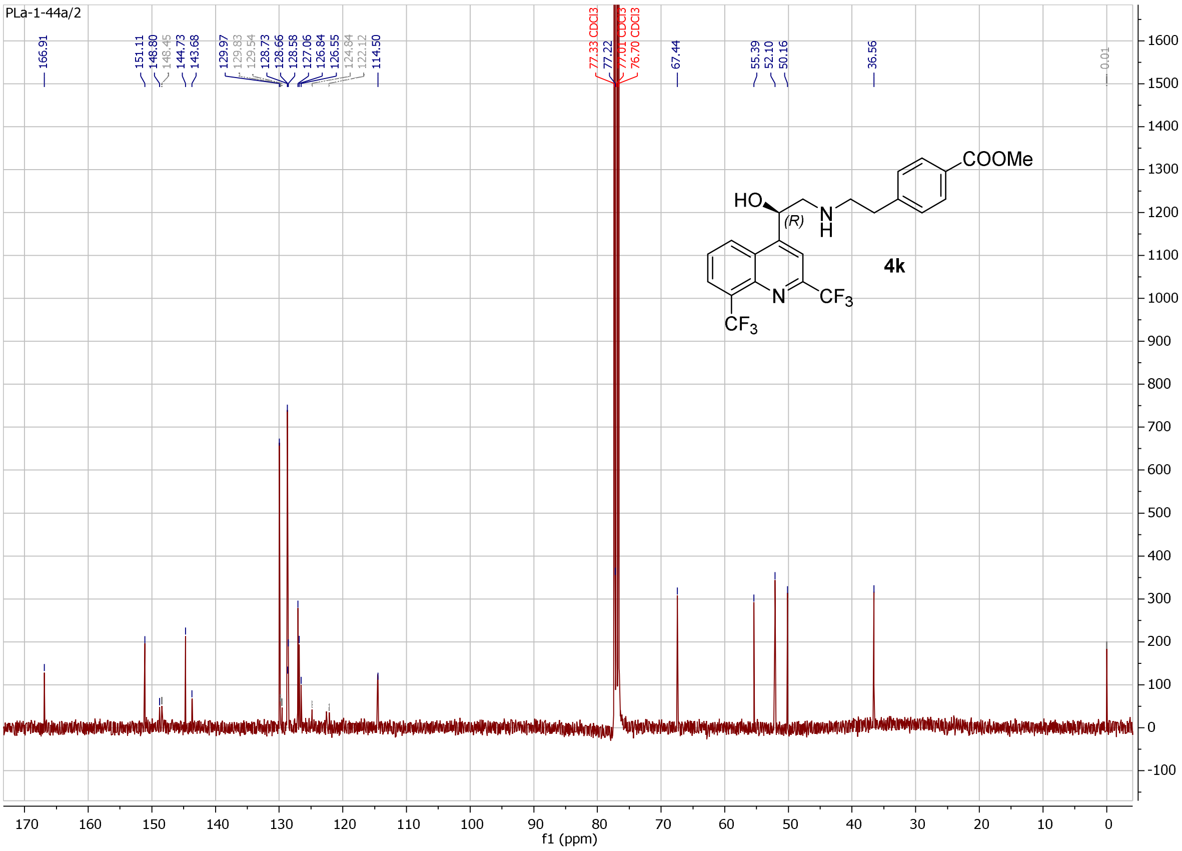

Figure S13: ¹H NMR of compound **4m** (400 MHz, Chloroform-*d*)

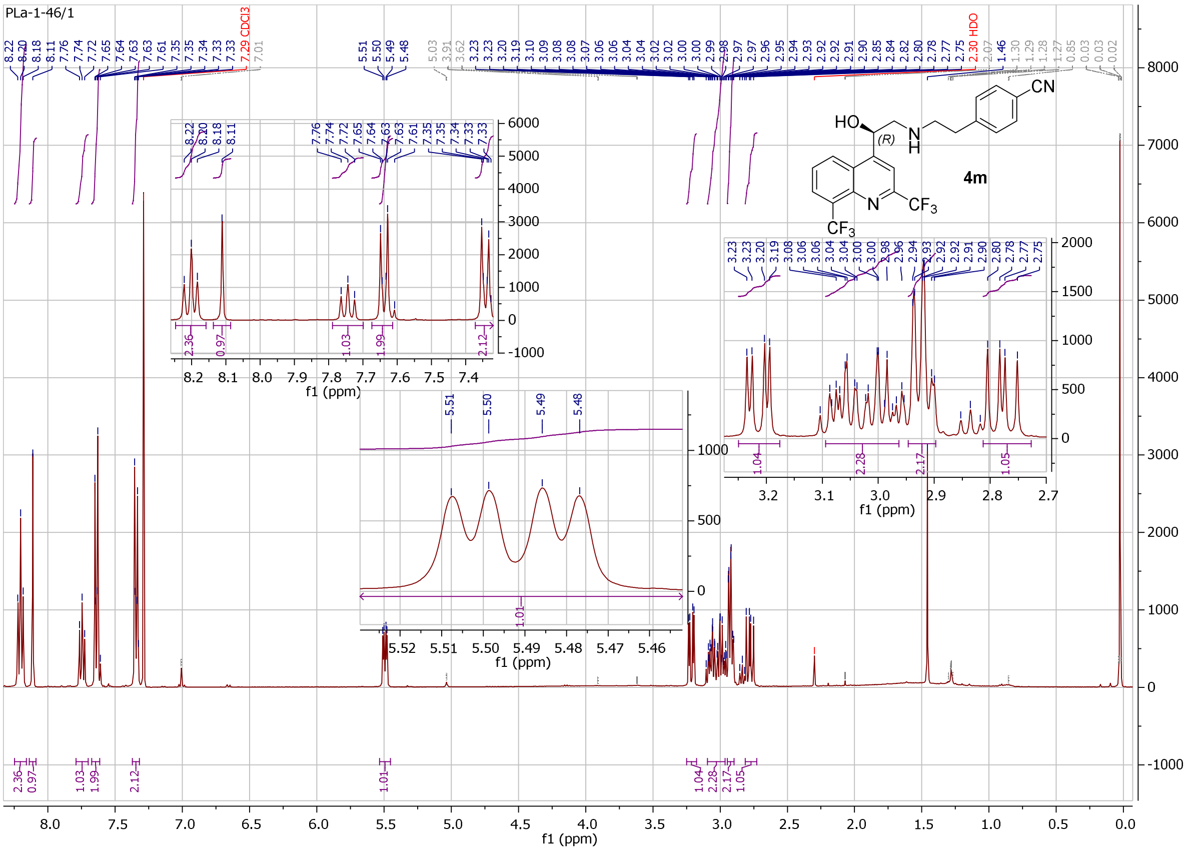

Figure S14: ¹³C NMR of compound **4m** (101 MHz, Chloroform-*d*)

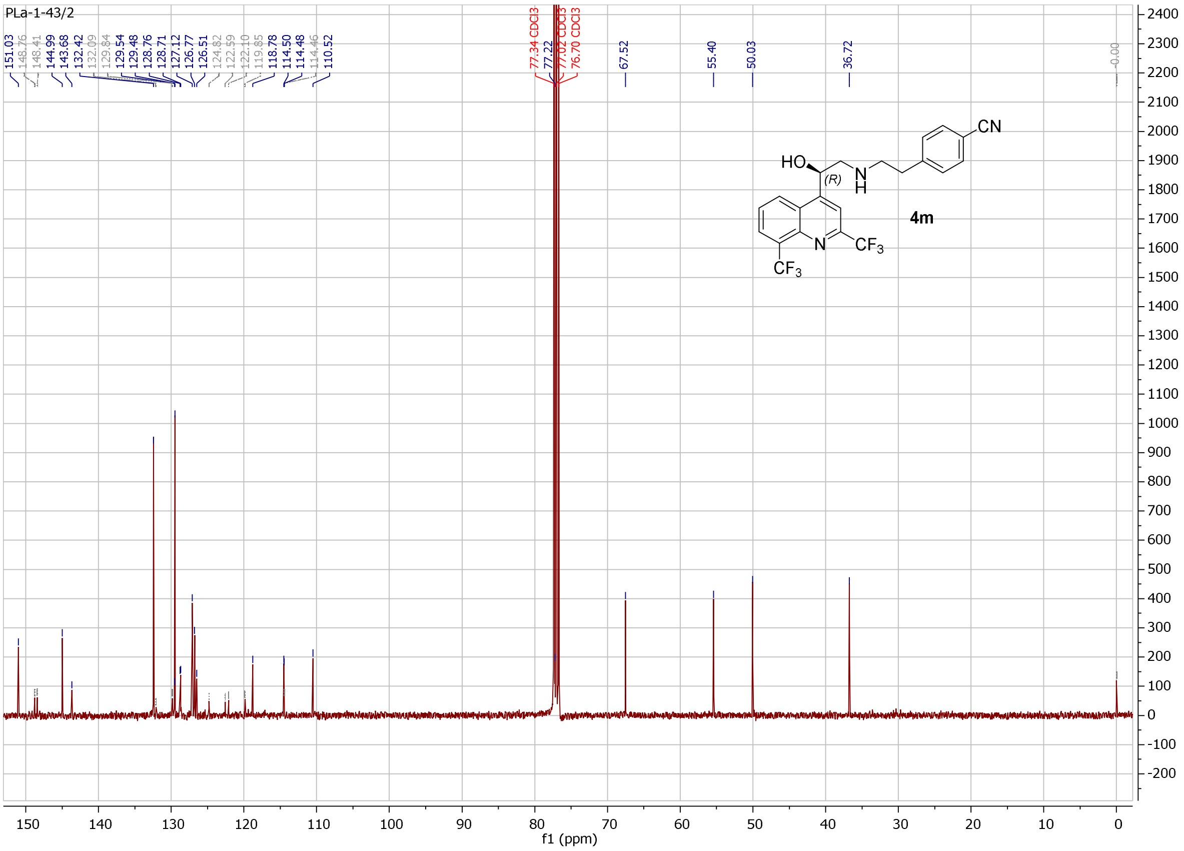

Figure S15: ^{1}H NMR of compound **4o** (400 MHz, Methanol-d_4)

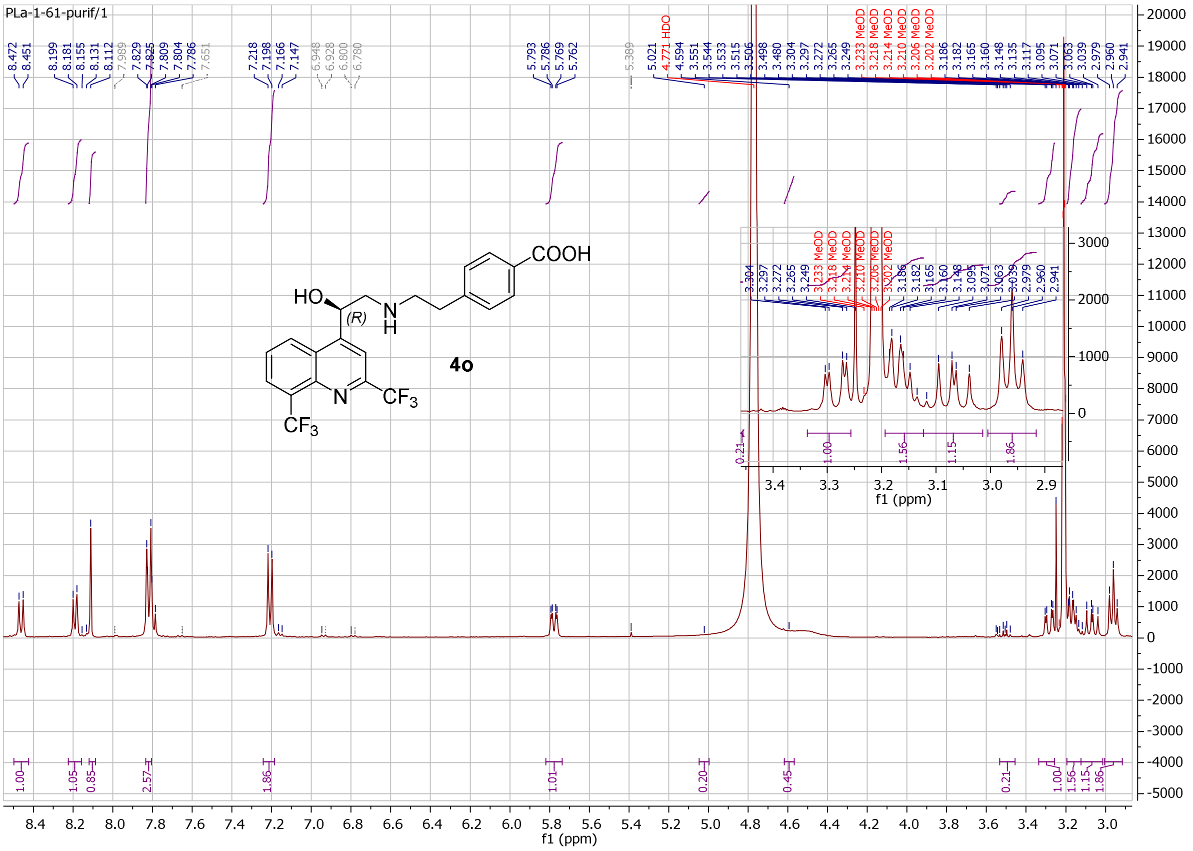

Figure S16: ^{13}C NMR of compound **4o** (101 MHz, Methanol-d_4)

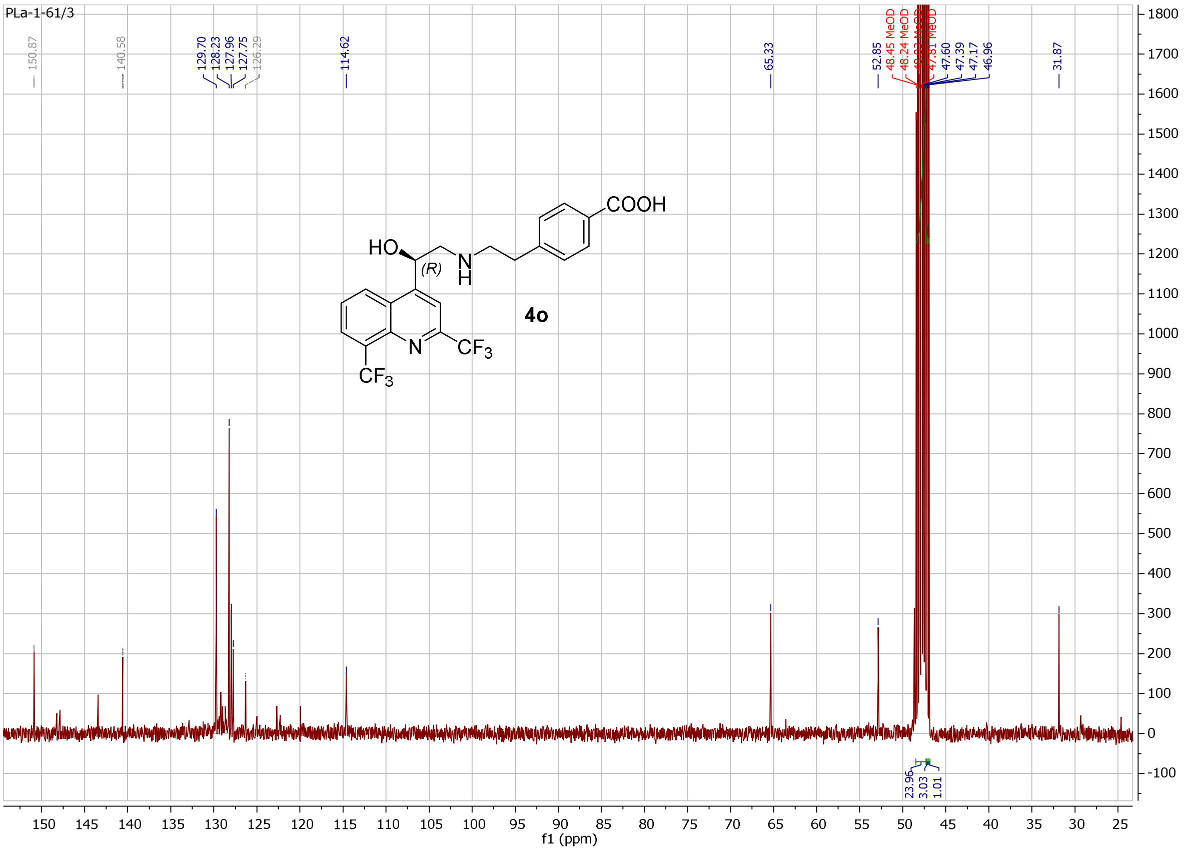

Figure S17: ^{1}H NMR of compound **4q** (400 MHz, Methanol-*d*4)

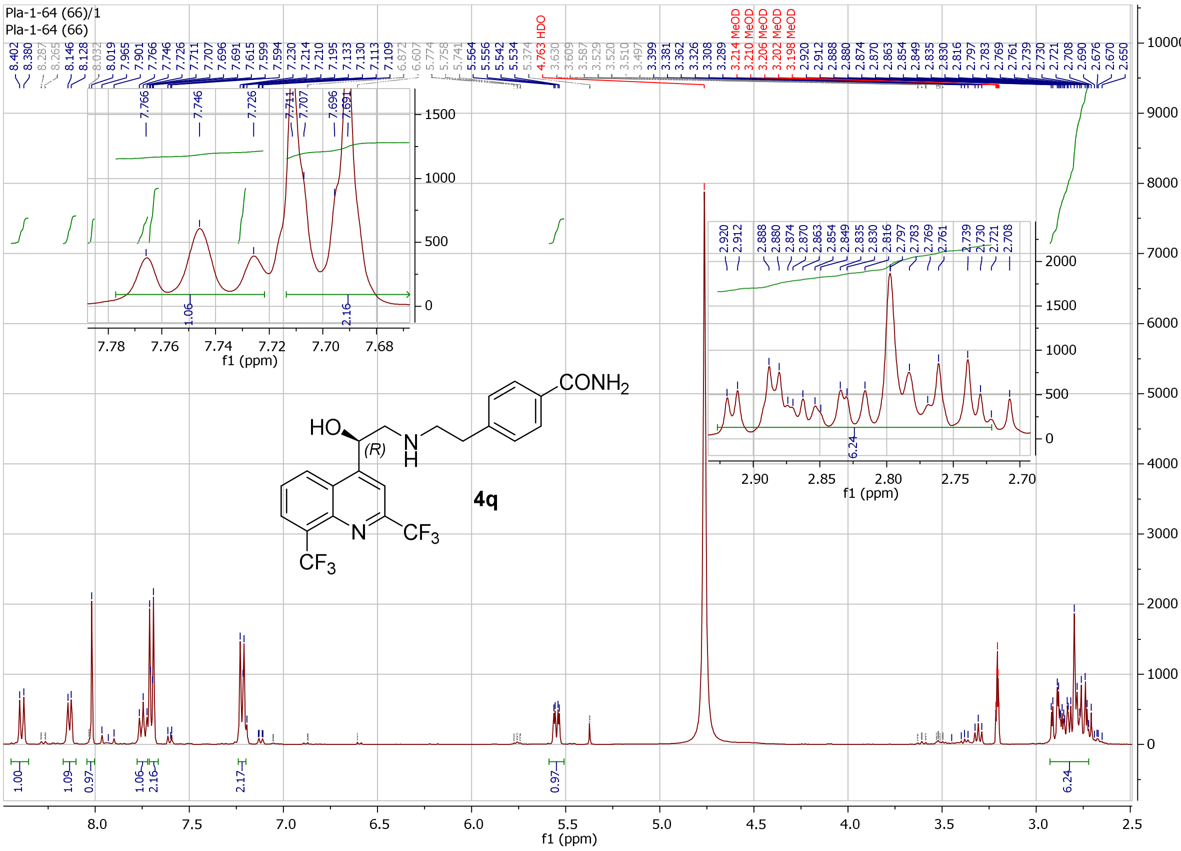

Figure S18: ^{13}C NMR of compound **4q** (101 MHz, Methanol-*d*4)

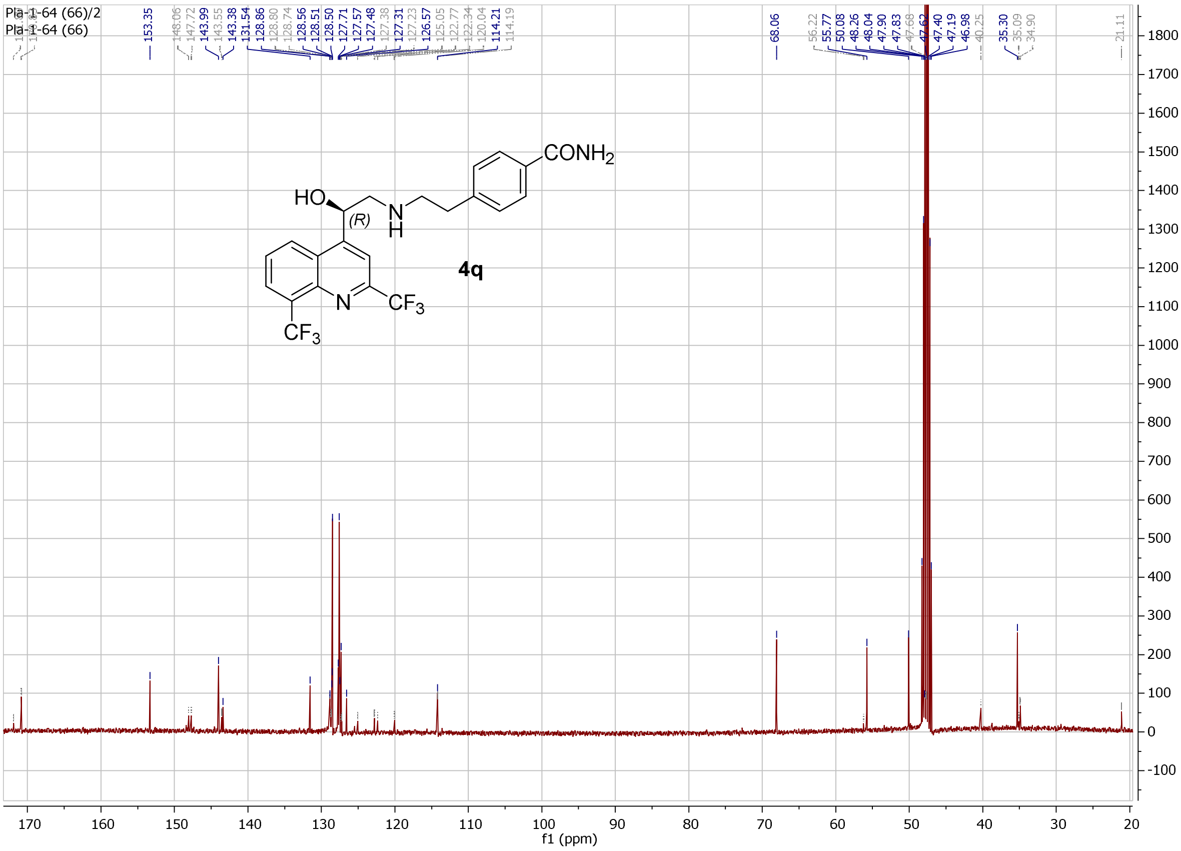

Figure S19: ^{1}H NMR of compound **4s** (400 MHz, Methanol-d_4)

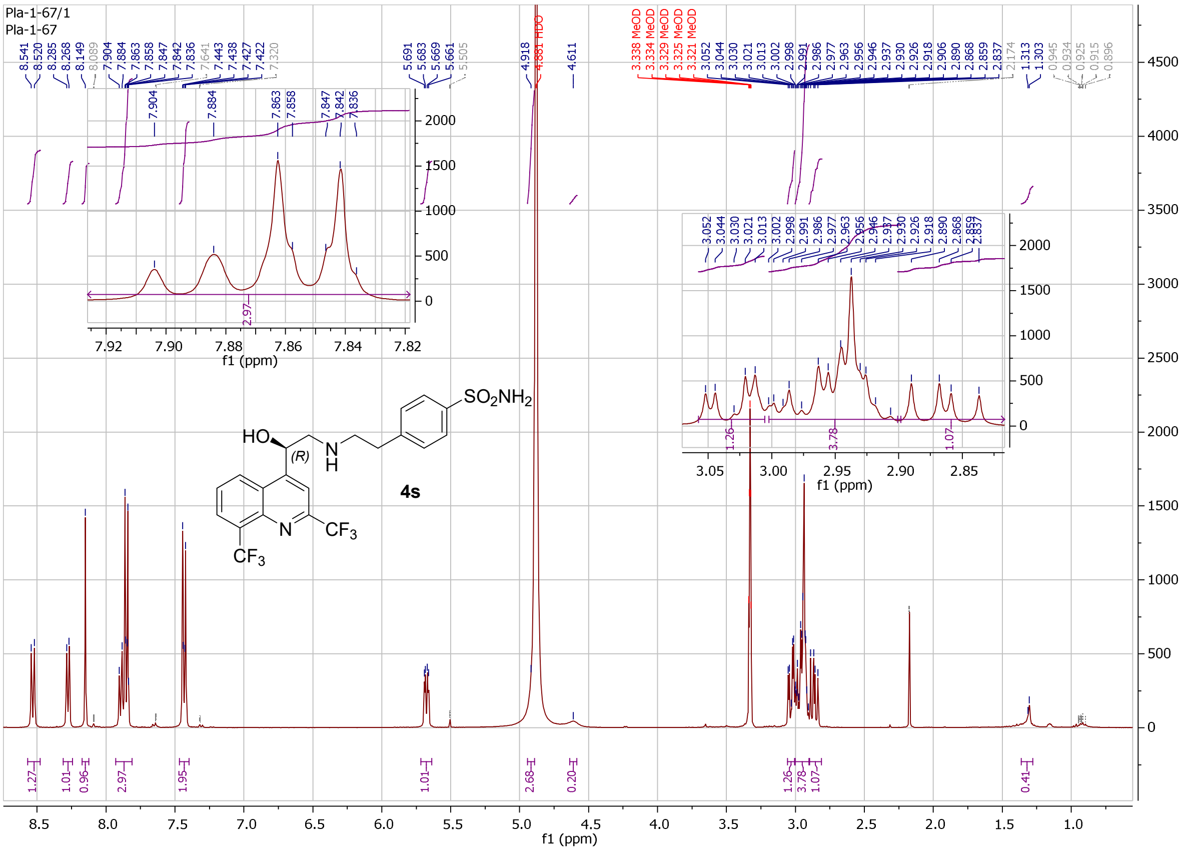

Figure S20: ^{13}C NMR of compound **4s** (101 MHz, Methanol-d_4)

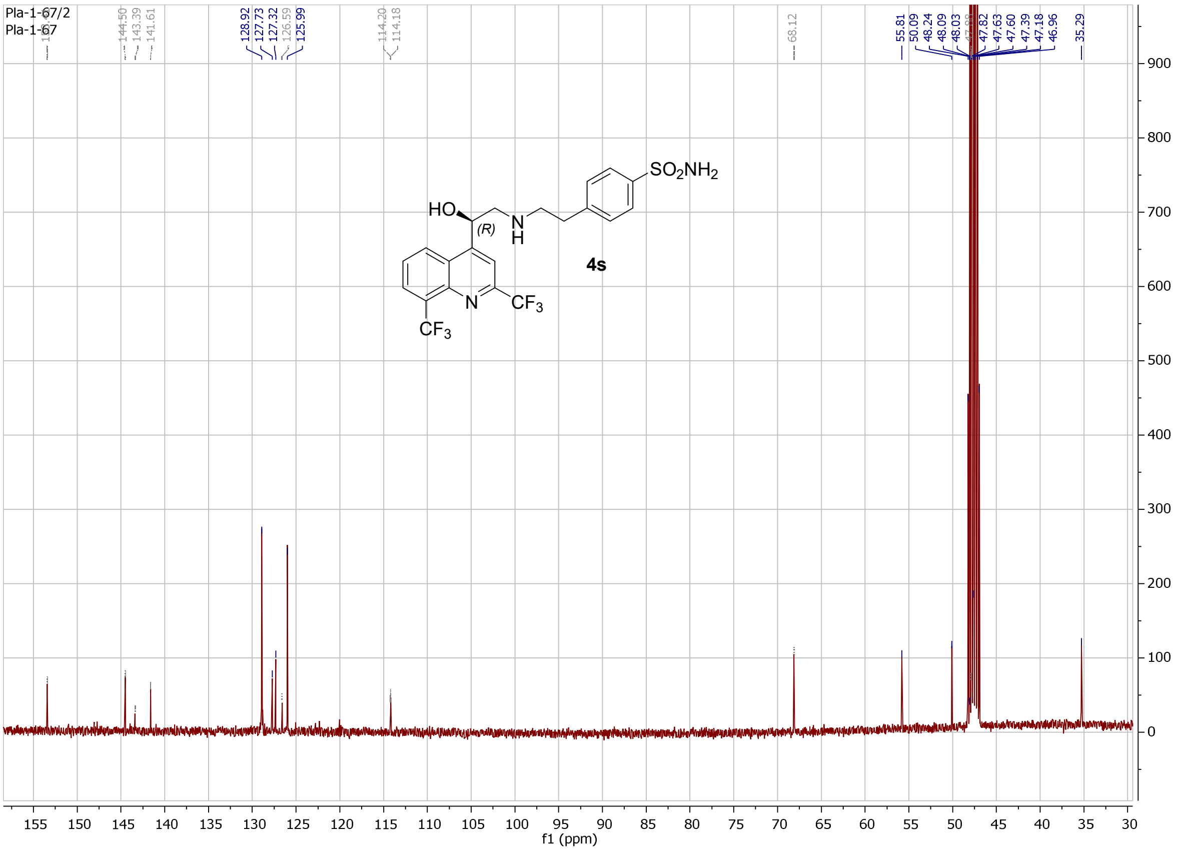

Figure S21: ^{1}H NMR of compound **4u** (400 MHz, Chloroform-*d*)

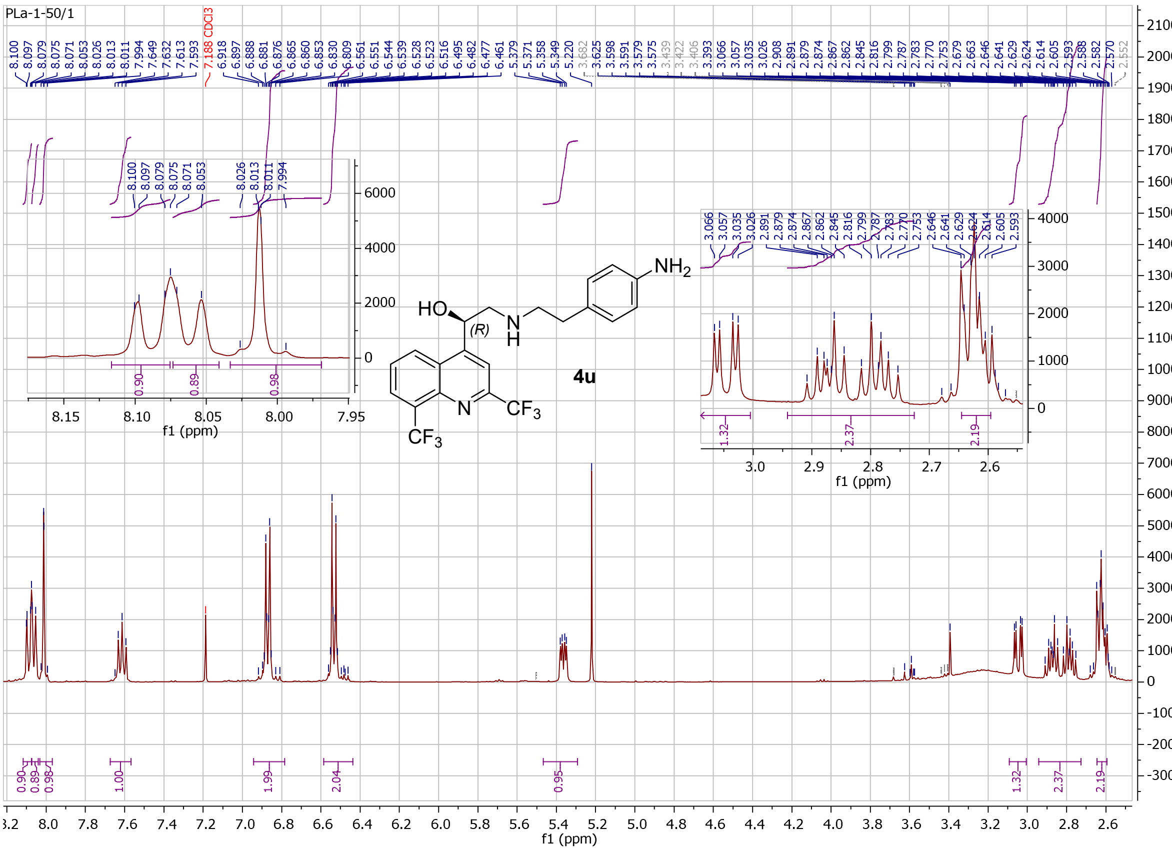

Figure S22: ^{13}C NMR of compound **4u** (101 MHz, Chloroform-*d*)

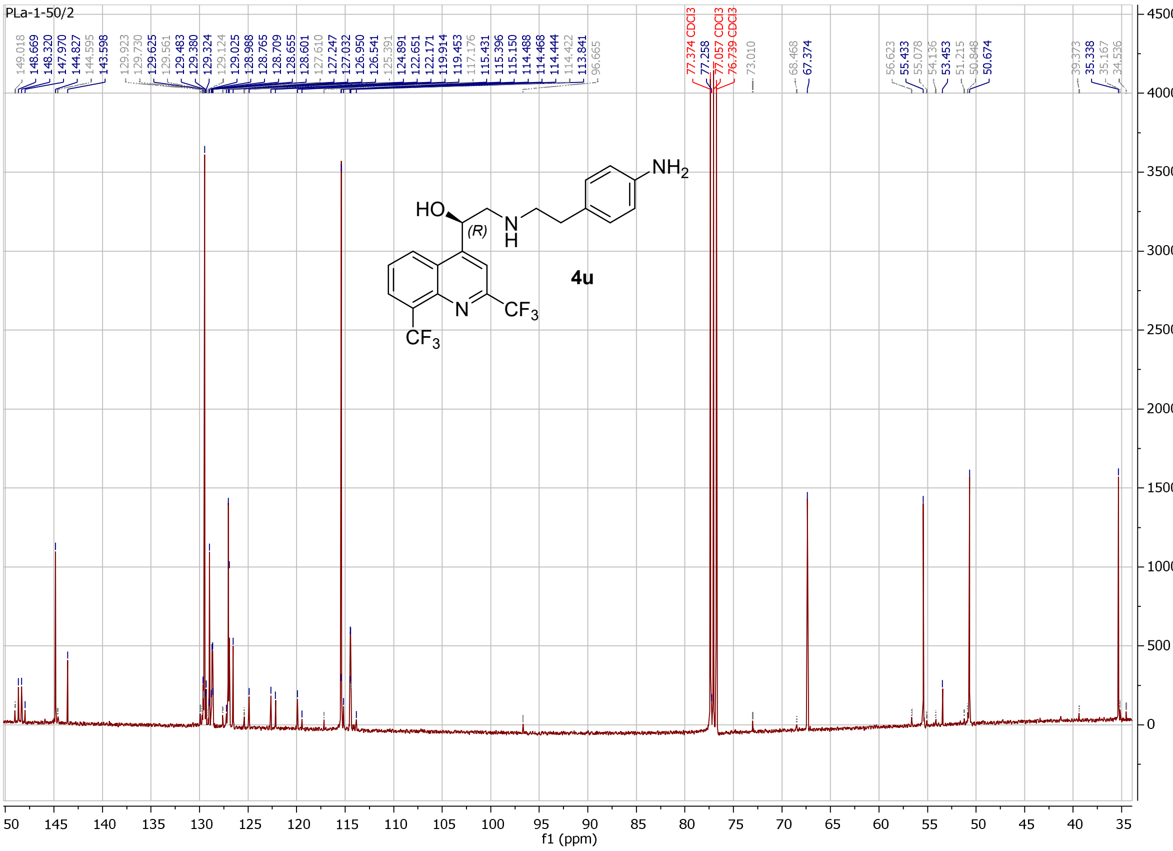

Supplement: Supplementary file 1 [file pharmaceuticals-12-00091-s001.pdf]
